# Global Review of the Age Distribution of Rotavirus Disease in Children Aged <5 Years Before the Introduction of Rotavirus Vaccination

**DOI:** 10.1093/cid/ciz060

**Published:** 2019-01-28

**Authors:** Mateusz Hasso-Agopsowicz, Chandresh Nanji Ladva, Benjamin Lopman, Colin Sanderson, Adam L Cohen, Jacqueline E Tate, Ximena Riveros, Ana Maria Henao-Restrepo, Andrew Clark, Global Rotavirus Surveillance Network and Rotavirus Age Study Collaborators, M Alkorta, C Atchison, S Banajeh, S Becker-Dreps, M Benhafid, N Bhandari, L Bodhidatta, T Braeckman, J Bwogi, R de Cassia Compagnoli Carmona, G Cilla, I Contreras-Roldan, B Coulson, N A Cunliffe, R Dagan, N Givon, J I Degiuseppe, S Dhiman, Z Dian, J Diaz, S Dutta, T Krishnan, B Manna, S Fletcher-Lartey, C Fu, D Gendrel, K S Ghenghesh, G Gonzalez Mago, S De Grazia, K Grimwood, M Groome, A Haque, U Heininger, E R Houpt, M Iturriza-Gomara, D Hungerford, C M Jarquin, J P McCracken, I L Contreras, C Cordon-Rosales, P Kaiser-Labusch, G Kang, S Kar, N Kiulia, K Kotloff, R Latipov, A Linhares, M Lorrot, M Mandile, C Mast, M A Mathew, F Matinon-Torres, J Matthijnssens, Z Mladenova, M Monini, M Montes, A Arana, M Motamedifar, A Najafi, T Nelson, J Nokes, F Ntoumi, K Numazaki, C O’Reilly, T J Ochoa, N A Page, A L Page, C Langendorf, A T Podkolzin, C Quach, M L Racz, A de Rougemont, G M Ruiz-Palacios, S K Saha, S Saha, S M Satter, L Soares, S M Sudarmo, K Shigemura, T Shirakawa, A F Athiyyah, B Tagbo, P Tarr, E Klein, D M Denno, A Turner, E B Uzoma, R R Vatosoa, E A Wandera, M Wikswo, D Payne, H Yhu-Chering, T Yoshikawa, K Sugata, Q Yuan, L Liying, K Zaman, X -N Zhou, S -X Zhang, W Xu, Fatima Serhan, Tomoka Nakamura, Sébastien Antoni, Mary Agócs, Jillian Murray, Thomas Cherian, Jason M Mwenda, Goitom Weldegebriel, Joseph N M Biey, Dah Cheikh, Nadia Teleb, Hossam Abdel Rahman, Hinda Ahmed, Danni Daniels, Dovile Videbaek, Annemarie Wasley, Simarjit Singh, Lucia de Oliveira, Gloria Rey-Benito, N Jennifer Sanwogou, Jayantha Liyanage, Pushpa Ranjan Wijesinghe, Nyambat Batmunkh, Varja Grabovac, Kimberley Fox, Fem Julia Paladin, Nicholas Henschke

**Affiliations:** 1 London School of Hygiene and Tropical Medicine, United Kingdom; 2 World Health Organization, Geneva, Switzerland; 3 Emory University, Atlanta, Georgia; 4 Centers for Disease Control and Prevention, Atlanta, Georgia

**Keywords:** vaccine, rotavirus, rotavirus gastroenteritis, age distribution

## Abstract

We sought datasets with granular age distributions of rotavirus-positive disease presentations among children <5 years of age, before the introduction of rotavirus vaccines. We identified 117 datasets and fit parametric age distributions to each country dataset and mortality stratum. We calculated the median age and the cumulative proportion of rotavirus gastroenteritis events expected to occur at ages between birth and 5.0 years. The median age of rotavirus-positive hospital admissions was 38 weeks (interquartile range [IQR], 25–58 weeks) in countries with very high child mortality and 65 weeks (IQR, 40–107 weeks) in countries with very low or low child mortality. In countries with very high child mortality, 69% of rotavirus-positive admissions in children <5 years of age were in the first year of life, with 3% by 10 weeks, 8% by 15 weeks, and 27% by 26 weeks. This information is critical for assessing the potential benefits of alternative rotavirus vaccination schedules in different countries and for monitoring program impact.

## Teaser

We analyzed the age distribution of children <5 years old with rotavirus gastroenteritis before the introduction of rotavirus vaccine. We found that the median age of rotavirus varies between 38 and 65 weeks and is negatively associated with child mortality.

Rotavirus gastroenteritis (RVGE) is estimated to cause approximately 200 000 child deaths each year [1]. More than half of the countries in the world now include live oral rotavirus vaccines in their national immunization programs [2]. There are 4 vaccines licensed for global use (Rotarix, GlaxoSmithKline; RotaTeq, Merck & Co; ROTAVAC, Bharat Biologicals; and ROTASIIL, Serum Institute of India), others for national use (eg, in Vietnam, China), and several others in the pipeline, including neonatal and nonreplicating injectable vaccines [3]. Rotavirus vaccines are currently coadministered with other vaccines that are already part of the routine immunization schedule. Most high-mortality countries use Rotarix, administered in 2 doses at 6 and 10 weeks of age. However, there is variation in the brand of vaccine used, as well as the target number of doses, target ages, and actual coverage and timeliness of each dose. The World Health Organization (WHO) recommends administering the first dose from 6 weeks of age, with an interval of at least 4 weeks between doses [4]. Randomized controlled trials have reported high vaccine efficacy (~90%) against severe RVGE in low-mortality countries but modest efficacy (~50%) in higher-mortality settings [5]. Alternative schedules are being considered to increase their impact. A neonatal vaccine has had promising results in Indonesia [6], and some studies have evaluated the potential of a booster dose given at around 9–12 months of age [7, 8]. Several studies and surveillance systems have collected information on RVGE age distributions, but much of it is unpublished or has been published in age bands that are too broad to allow a detailed assessment of the potential impact of alternative rotavirus vaccination schedules. More granular age distributions would also help to quantify the number of RVGE cases expected to occur at specific ages, so that changes can be monitored after vaccination. More generally, there is a need to update the global evidence on RVGE age distributions, compare them between countries and regions, and establish a reliable method for extrapolating them to countries without data. An unpublished review was conducted in 2012 [9], but this did not include the large multicountry Global Rotavirus Surveillance Network (GRSN) database [10], and several pivotal multicountry studies have also been published since [11–13].

In this article, we aim to estimate granular age distributions of rotavirus disease outcomes in children aged <5 years by type of RVGE presentation, country, and mortality level, before the introduction of rotavirus vaccines. This article does not generate estimates of the potential impact of alternative rotavirus vaccination schedules but does provide inputs that will be important for those calculations.

## METHODS

### Ethical Approval

This study was approved by the ethical committee of the London School of Hygiene and Tropical Medicine (ethics reference number 14398). All authors and countries gave their consent to analyze and publish the data.

### Search Strategy and Study Selection

We sought country datasets containing counts of rotavirus-positive disease in children aged <5 years before the introduction of rotavirus vaccines. A country dataset is defined as a dataset derived from a single study (eg, hospital surveillance, case-control, cohort) within a single country, reporting on a single rotavirus-positive outcome/presentation (community cases, clinic visits, emergency visits, hospitalizations, deaths). If a dataset contained multiple subnational locations and/or multiple calendar years, then these were aggregated, and any relevant exclusion criteria were applied to the aggregated dataset. When studies reported multiple rotavirus-positive presentations, each presentation was considered to be a distinct dataset. Prevaccine datasets only included data for years prior to rotavirus vaccine introduction.

First, we analyzed the WHO GRSN database, which contains information about hospital admissions among children aged <5 years from surveillance sites in 69 countries [10]. In these sites, rotavirus positivity is determined by enzyme immunoassay (EIA). We applied the definition described above and aggregated subnational locations and multiple calendar years to create unique prevaccine introduction GRSN country datasets. The year of rotavirus vaccine introduction was determined by WHO/United Nations Children’s Fund (UNICEF) estimates of national immunization coverage [14]. If a country dataset did not have data on hospital admissions by day of age, then we used month of age. Admissions recorded as aged zero days were removed for face validity (inconsistent with the rotavirus incubation period).

Second, we conducted a systematic literature review adhering to the Preferred Reporting Items for Systematic Reviews and Meta-analyses (PRISMA) guidelines to identify other relevant rotavirus studies. A full description of the search strategy is provided in the Supplementary Appendix. In brief, we searched for papers published between January 1990 and February 2017 and publications in English, French, Spanish, and Polish. We excluded studies in which rotavirus positivity was not determined by EIA or quantitative polymerase chain reaction; nonhuman studies; nosocomial infection studies; studies without information on individuals aged <5 years; special populations such as human immunodeficiency virus–infected patients; meta-analyses and systematic reviews reporting regional or global age distributions; and papers without an accessible full-text link. Two independent reviewers (M. H.-A., C, N. L.) screened abstracts and any ambiguity was resolved by a third reviewer (A. D. C.). A letter was sent by email to the investigators of all studies identified in the systematic review. Investigators were asked to provide anonymized data or complete a standard data extraction table with counts by week of age up to 5.0 years. If the investigators did not respond before the end of August 2017 and no other study was available for that country, we extracted the age distribution reported in the publication. We included all country datasets that were obtained from a previously unpublished literature and database search conducted by Sanderson et al in 2012 [9]. This included articles published between 1990 and 2011.

All country datasets were combined into a central database with a standard format and list of variables and analyzed together with the GRSN datasets. We cross-checked datasets identified through the literature search and GRSN to avoid data duplication. Prior to analyzing the datasets, we excluded studies that included <35 RVGE events, had known concerns about EIA quality, had <3 age bands <1 year of age, and did not capture cases from birth. We designed a tool to assess the risk of bias in randomized controlled trials and observational studies and assigned very low, low, or medium risk of bias to each country dataset. The risk of bias was scored against a list of 5 criteria (Supplementary Appendix).

### Data Analysis

We fit a range of parametric distributions (gamma, Weibull, log-normal, log-logistic, Burr) to several GRSN datasets that were reported by day of age, and that represented the extreme range of younger and older age distributions globally. We fit age distributions using maximum likelihood estimation. The best-fitting distribution was chosen by comparing goodness-of-fit statistics (Kolmogorov-Smirnov, Cramer–von Mises, Anderson-Darling) and goodness-of-fit criteria (Akaike information criterion, Bayesian information criterion). For each country dataset, we calculated the best-fitting parameters of the chosen distribution. We generated summary tables with the median age, interquartile age range, and the cumulative proportion of RVGE cases aged <5 years that were estimated to occur at different granular ages between birth and age 5.0 years. We reported the root mean squared error and mean absolute error for the parametric distribution fitted to each country dataset. All analyses were conducted using R version 3.4.1 using the following packages: polspline, nloptr, zoo, MASS, fitdistrplus, actuar, and mutil.

We assigned each of the 201 countries in the world to an under-5 mortality quintile (very low, low, medium, high, and very high) using 2010–2015 estimates of under-5 mortality as reported by the United Nations Population Division 2017 revision [15]. We grouped all datasets according to the under-5 mortality quintile of the country concerned, and calculated the median age and median best-fitting parameters for each stratum. We also ran a series of regression analyses to explore which combinations of variables would best predict the median age and parameters of the chosen parametric distribution. To compare differences in rotavirus disease presentations, we plotted the full set of median ages reported for a given presentation against their respective 2010–2015 under-5 mortality rates. We fit a least-squares line of best fit for each presentation, reported the *R*^2^ value, and compared the best-fitting lines.

We used ArcGIS mapping software to display the median age of rotavirus hospitalization estimated for each country in the world. If more than a single dataset was available for a country, we calculated the median age and median best-fitting parameters of all datasets for that country. If no dataset was available, we assigned the median age of the country’s corresponding mortality stratum.

## RESULTS

**Figure 1.**
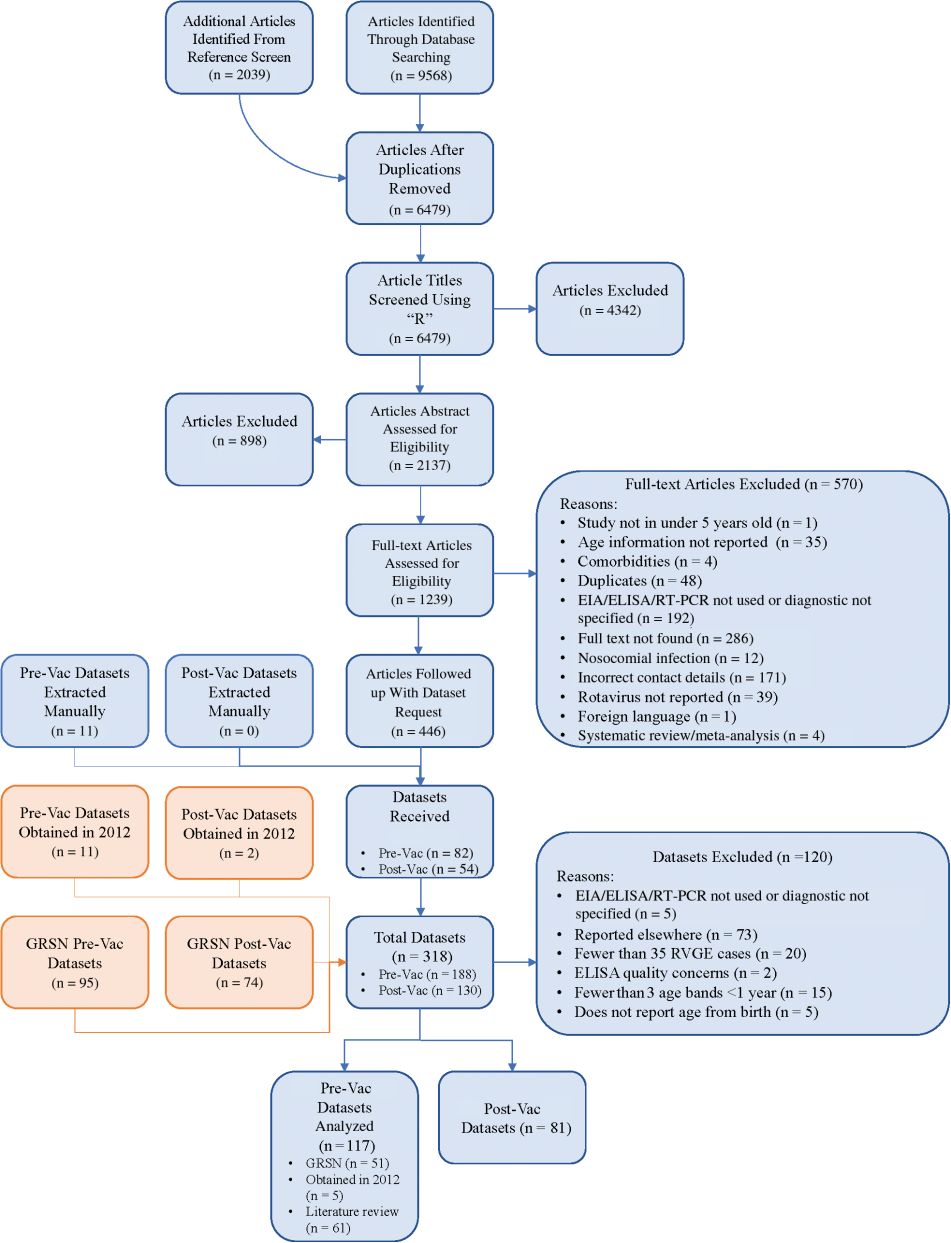
Flowchart of the study selection process and data extraction. Some studies contained multiple datasets. Abbreviations: EIA, enzyme immunoassay; ELISA, enzyme-linked immunosorbent assay; GRSN, Global Rotavirus Surveillance Network; Pre-Vac, prevaccination; Post-Vac, postvaccination; RT-PCR, reverse-transcription polymerase chain reaction.

We identified 117 prevaccination datasets with rotavirus-positive events among children <5 years of age (6 datasets with community cases, 12 with clinic visits, 7 with emergency visits, 92 with hospital admissions, and 0 with deaths) (Table 1; Figure 1). Around half of the country datasets (51/117) were rotavirus-positive cases identified through hospital-based sentinel site surveillance from the GRSN (35 reported by day of age and 16 reported by month of age). The other half (66/117) were identified from the systematic literature review (n = 61) or obtained from the previously unpublished review (n = 5). The 117 prevaccination datasets were taken from 47 studies with very low (n = 24), low (n = 12), and medium (n = 11) risk of bias.

**Table 1.** Number of Country Datasets Containing Rotavirus Gastroenteritis Age Distributions Before the Introduction of Rotavirus Vaccination, by Type of Presentation and Under-5 Mortality Quintile

Quintile for 2010–2015 Under-5 Mortality Rate	No. of Country Datasets (No. of Rotavirus-positive Cases)				
	Hospital Admissions	Emergency Visits	Clinic Visits	Community Cases	Total
Very low	13 (31 211)	3 (10 467)	3 (1552)	0 (0)	19 (43 230)
Low	8 (10 348)	2 (179)	1 (41)	0 (0)	11 (10 568)
Medium	14 (13 990)	0 (0)	1 (224)	1 (89)	16 (14 303)
High	31 (23 557)	2 (167)	3 (461)	4 (536)	40 (24 721)
Very high	26 (26 142)	0 (0)	4 (1066)	1 (71)	31 (27 279)
Total	92 (105 248)	7 (10 813)	12 (3344)	6 (696)	117 (120 101)

**Figure 2.**
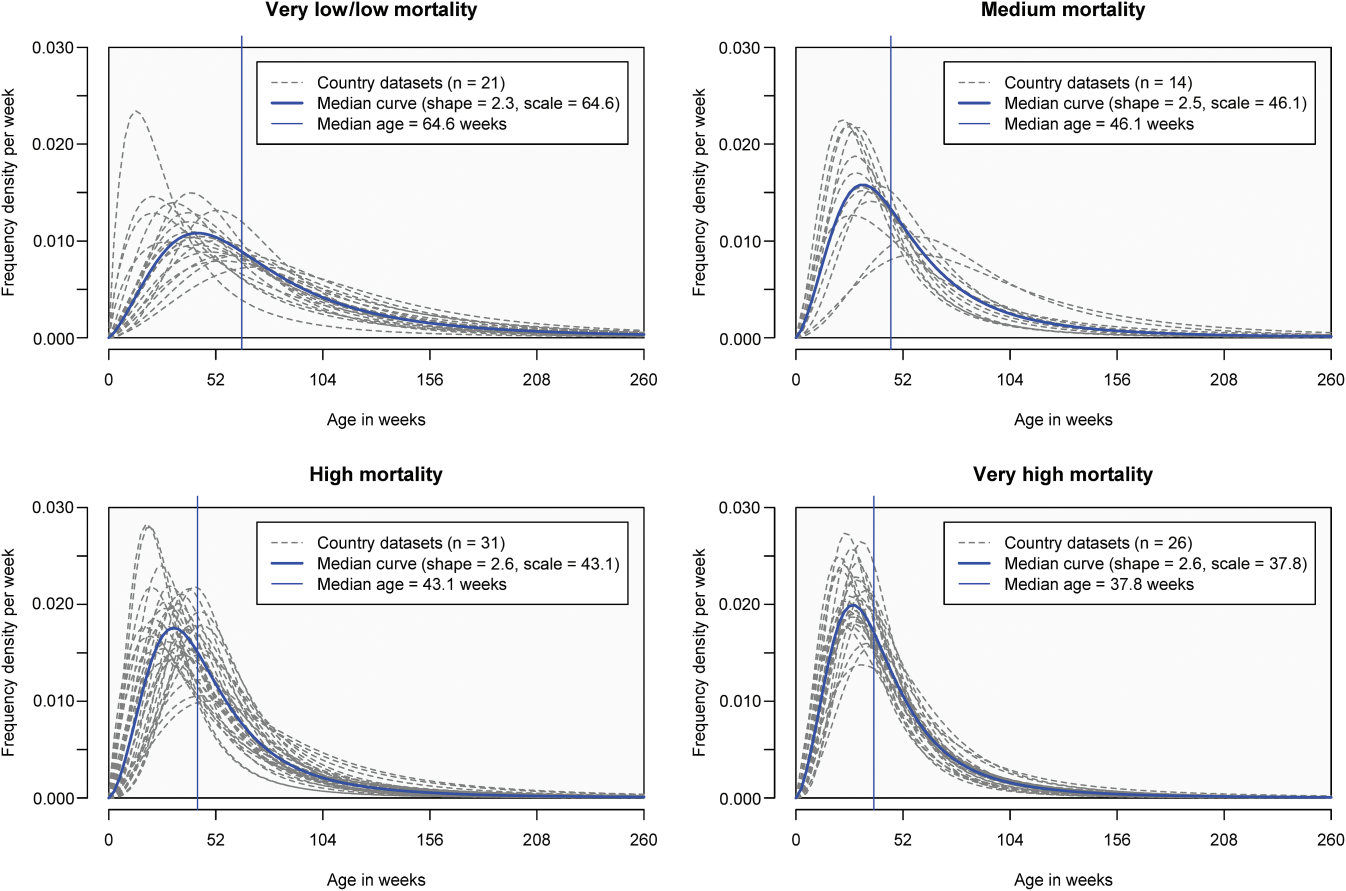
Age distributions of rotavirus-positive hospital admissions by under-5 mortality strata.

Log-logistic age distributions had favorable goodness-of-fit statistics and criteria (Supplementary Figures 1 and 2), so were used to generate summary statistics on the age distribution of hospital admissions aged <5 years (Supplementary Tables 2 and 3). The median age of RVGE hospital admission was 38 weeks (interquartile range [IQR], 25–58 weeks) in countries with very high child mortality, 43 weeks (IQR, 28–68 weeks) in countries with high child mortality, 46 weeks (IQR, 29–72 weeks) in countries with medium child mortality, and 65 weeks (IQR, 40–107 weeks) in countries with low/very low child mortality (Figure 2). We collapsed the low and very low child mortality strata because they had a similar median age (67 weeks for very low and 63 weeks for low) and regression analyses showed there was no difference between the 2 strata (*P* = .234; Supplementary Table 5, regression model 5). In countries with very high child mortality, 69% of rotavirus-positive admissions in children aged <5 years were in the first year of life, with 3% by 10 weeks, 8% by 15 weeks, and 27% by 26 weeks. There was considerable variation within each child mortality stratum. For example, in the very high child mortality stratum, the median age ranged from 29 weeks (IQR, 19–46 weeks) in Zambia to 50 weeks (IQR, 30–81 weeks) in Ethiopia. Similarly, in the low/very low mortality stratum, the median age ranged from 35 weeks (IQR, 19–64 weeks) in France to 101 weeks (IQR, 65–157 weeks) in Ukraine.

**Figure 3.**
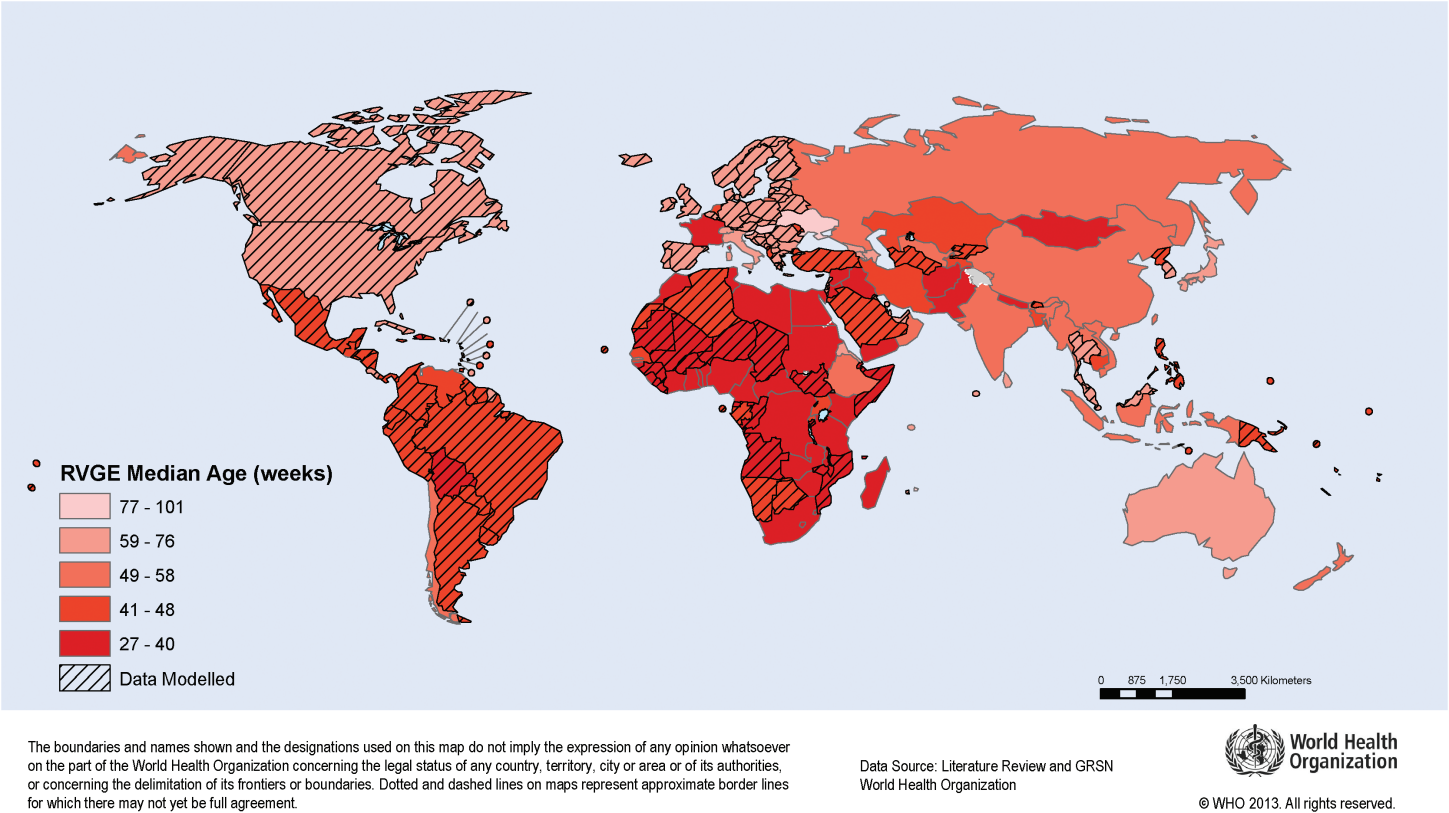
Estimated and extrapolated prevaccination median age of rotavirus-positive hospital admissions in children aged <5 years, by country. Lighter red represents younger median age and darker red represents older median age. If >1 study was conducted within a country, the median of median ages was used. If no data were available for a country, the median age was extrapolated (indicated by diagonal shading) using the median age of the under-5 mortality stratum. Abbreviations: GRSN, Global Rotavirus Surveillance Network; RVGE, rotavirus gastroenteritis. The map is reprinted with permission from the World Health Organization.

Globally, most countries with a low median age were in Africa (Figure 3). In general, the median age of rotavirus-positive hospital admissions decreased as child mortality increased (one-way analysis of variance; *P* < .0001), but there were notable outliers such as France and the Netherlands, where the median age was exceptionally low (35 and 48 weeks, respectively), and Mauritius and Ukraine, where the median age was exceptionally high (84 and 101 weeks, respectively). Regression models with more variables provided no substantive advantage over the simple stratification by under-5 mortality quintile (Supplementary Tables 4 and 5).

There were relatively few global datasets with age distributions for community cases, clinic visits, and emergency visits, and none for RVGE deaths that met our inclusion criteria. The median age for RVGE emergency visits was around 10 weeks younger than the median age for RVGE hospital admissions. The median age for RVGE clinic visits was around 5 weeks older than the median age for RVGE hospital admissions (Supplementary Tables 6–8). This pattern was consistent across settings with different under-5 mortality rates (Supplementary Figure 3).

## DISCUSSION

We have gathered and synthesized a large amount of evidence on rotavirus age distributions globally. To our knowledge, this is the first systematic global study to estimate granular age distributions by country, mortality stratum, and level of care sought. We use statistically robust and standard methods to provide reproducible parametric age distributions for each country. We show that the median age of rotavirus disease varies between and within countries but tends to occur at a much younger age in higher-mortality settings. According to the basic principles of infectious disease dynamics, a younger average age of infection is likely to be associated with a higher force of infection. This is consistent with reported incidence rates of rotavirus infection, which have been shown to peak at 5.5 months in Vellore, India (high mortality) and at 20 months in Mexico City (medium mortality). However, in these sites the overall rate of infection and the age distribution of symptomatic RVGE cases were not substantially different [16]. This is probably because infections among Indian children were less likely to protect against subsequent disease, leading to several cases in older Indian children [17, 18]. This is consistent with the lower protection acquired from doses of rotavirus vaccination in higher-mortality settings [19]. Our analysis of a much larger number of settings has shown that the most severe RVGE cases (ie, those being admitted to hospital) tend to occur at younger ages in higher-mortality settings. We hypothesize that this is probably due to a higher force of infection and shorter intervals between repeat infections. There could also be important age-specific differences in the early management and treatment of RVGE in higher-mortality settings.

Our analysis relies heavily on the WHO GRSN database, which may include sentinel sites that are not fully representative of the country concerned. Importantly, healthcare-seeking behavior varies by country and age, and this may help to explain heterogeneities observed within each mortality stratum. For example, in some settings with high rates of private healthcare, children aged <1 year may be more likely to be treated outside of the regular sentinel surveillance system. In Hungary, Slovenia, and Ukraine, the median age of rotavirus-positive hospital admissions was 86, 88, and 101 weeks, respectively. This high median age might simply be a characteristic of rotavirus in Central and Eastern Europe or may reflect other surveillance peculiarities (eg, underrecruitment of younger patients or overrecruitment of mild RVGE cases). We analyzed the very low and low mortality strata without Ukraine, but that did not change the median age of 65 weeks. In other datasets, there may be a bias to younger ages. For example, we found a surprisingly low median age of hospital admission from multiple datasets in France (median age, 27–41 weeks) for reasons that are not clear.

We chose to fit parametric distributions rather than report the actual age distributions observed in each study. This required an assumption to be made about the standard functional form of the distribution. However, our parametric fitting approach (1) provides a function that can be easily reproduced by others; (2) avoids the issue of heaping—that is, the tendency to report cases at exactly 1.0 years, 2.0 years, etc, an issue that has been evident in many of the datasets because of a reporting artefact; (3) smooths distributions based on small (noisy) samples; and (4) allows standard reporting of the proportion of RVGE cases that occur at specific ages, for example, the proportion of cases occurring before the first dose of rotavirus vaccine at 6 weeks, or before vaccine age restrictions are applied at 15 weeks. We also explored nonparametric smoothing approaches. We used kernel density estimation, with default Gaussian smoothing. However, heaping was evident in some of the datasets, and areas of density below zero were common. One way to avoid this is to truncate the density at zero, but this introduces a bias in the distribution and creates an implausible cliff-edge at zero in some datasets. Another way to avoid this is to use logspline density estimation, with the lower bound set to zero. This worked well for some datasets but not others and required manual adjustment to the number and location of knots, so it was not practical as a standardized approach.

We obtained many datasets on rotavirus-positive hospital admissions but few on other presentations. No datasets with rotavirus-positive deaths met our inclusion criteria because they had <35 deaths, and it was very difficult to ascertain whether the deaths were entirely attributable to rotavirus. Compared to hospital admissions, we found a higher median age for clinic visits and a younger median age for emergency visits, but this was based on very few data points and more data would be needed to confirm this.

In conclusion, the median age of rotavirus disease in children aged <5 years varies between and within countries but tends to be younger in higher-mortality settings. The age distributions presented in this article should provide information that is critical for assessing the potential benefits of alternative rotavirus vaccination schedules in different countries, and for monitoring the impact of rotavirus vaccines.

## Supplementary Data

Supplementary materials are available at *Clinical Infectious Diseases* online. Consisting of data provided by the authors to benefit the reader, the posted materials are not copyedited and are the sole responsibility of the authors, so questions or comments should be addressed to the corresponding author.

ciz060_suppl_Supplementary_Material

## Notes

***Acknowledgments.* **The authors thank the numerous Rotavirus Age Study Collaborators who generously shared more granular data than was available in the published literature: M. Alkorta; C. Atchison; S. Banajeh; S. Becker-Dreps; M. Benhafid; N. Bhandari; L. Bodhidatta; T. Braeckman; J. Bwogi; R. de Cassia Compagnoli Carmona; G. Cilla; I. Contreras-Roldan; B. Coulson; N. A. Cunliffe; R. Dagan and N. Givon; J. I. Degiuseppe; S. Dhiman; Z. Dian; J. Diaz; S. Dutta, T. Krishnan, and B. Manna; S. Fletcher-Lartey; C. Fu; D. Gendrel; K. S. Ghenghesh; G. Gonzalez Mago; S. De Grazia; K. Grimwood; M. Groome; A. Haque; U. Heininger; E. R. Houpt; M. Iturriza-Gomara, D. Hungerford, and the EuroRotaNet Network; C. M. Jarquin, J. P. McCracken, I. L. Contreras and C. Cordon-Rosales; P. Kaiser-Labusch; G. Kang; S. Kar; N. Kiulia; K. Kotloff; R. Latipov; A. Linhares; M. Lorrot; M. Mandile; C. Mast and Merck & Co; M. A. Mathew; F. Matinon-Torres; J. Matthijnssens; Z. Mladenova; M. Monini; M. Montes; A. Arana; M. Motamedifar; A. Najafi; T. Nelson; J. Nokes; F. Ntoumi; K. Numazaki; C. O’Reilly; T. J. Ochoa; N. A. Page; A. L. Page; C. Langendorf; A. T. Podkolzin; C. Quach; M. L. Racz; A. de Rougemont; G. M. Ruiz-Palacios; S. K. Saha; S. Saha; S. M. Satter; L. Soares; S. M. Sudarmo, K. Shigemura, T. Shirakawa; A. F. Athiyyah; B. Tagbo; P. Tarr, E. Klein; D. M. Denno; A. Turner; E. B. Uzoma; R. R. Vatosoa; E. A. Wandera; M. Wikswo, D. Payne and the Centers for Disease Control and Prevention (CDC); H. Yhu-Chering; T. Yoshikawa; K. Sugata; Q. Yuan; L. Liying; K. Zaman; X.-N. Zhou, S.-X. Zhang, and W. Xu. They thank the various contributors to the World Health Organization (WHO) Global Rotavirus Surveillance Network: Fatima Serhan, Tomoka Nakamura, Sébastien Antoni, Mary Agócs, Jillian Murray, Thomas Cherian (WHO headquarters); Jason M. Mwenda, Goitom Weldegebriel, Joseph N. M. Biey, Dah Cheikh (WHO Regional Office for Africa); Nadia Teleb, Hossam Abdel Rahman, Hinda Ahmed (WHO Regional Office for the Eastern Mediterranean); Danni Daniels, Dovile Videbaek, Annemarie Wasley, Simarjit Singh (WHO Regional Office for Europe); Lucia de Oliveira, Gloria Rey-Benito, N. Jennifer Sanwogou (Pan American Health Organization/WHO Regional Office for the Americas); Jayantha Liyanage, Pushpa Ranjan Wijesinghe (WHO Regional Office for South-East Asia); and Nyambat Batmunkh, Varja Grabovac, Kimberley Fox, Fem Julia Paladin (WHO Regional Office for the Western Pacific). They also thank Nicholas Henschke and the Cochrane Response Group for sharing their expertise about assessing bias in observational studies.

***Financial support.* **This work was supported by the Bill & Melinda Gates Foundation (BMGF), Grant Numbers OPP1147721 and OPP1163272. Support for this project was also provided by PATH, WHO and NIH (R01 AI112970).

***Potential conflicts of interest.* **B. L. has received personal fees from Takeda Pharmaceuticals and the CDC Foundation. All other authors report no potential conflicts. All authors have submitted the ICMJE Form for Disclosure of Potential Conflicts of Interest. Conflicts that the editors consider relevant to the content of the manuscript have been disclosed.
