# Supplementary material for: Global Review of the Age Distribution of Rotavirus Disease in Children Aged <5 Years Before the Introduction of Rotavirus Vaccination
Source: Clin Infect Dis. 2019 Jan 28;69(6):1071–8. doi: 10.1093/cid/ciz060 (PMC6736387; doi:10.1093/cid/ciz060)
Supplement: ciz060_suppl_Supplementary_Material [file ciz060_suppl_supplementary_material.docx]

**Appendix to:**

**Global review of the age distribution of rotavirus disease in children aged <5 years before the introduction of rotavirus vaccination.**

Mateusz Hasso-Agopsowicz, Chandresh Nanji Ladva, Benjamin Lopman, Colin Sanderson, Adam L. Cohen, Global Rotavirus Surveillance Network, Rotavirus Age Distribution Collaborators, Jacqueline E. Tate, Ximena Riveros, Ana Maria Henao-Restrepo, Andrew Clark

**Search strategy**

1. **Search Terms**
   1. rotavir*
   2. Rotavirus Infections/ or Rotavirus
   3. 1 or 2
   4. incidence
   5. hospital*
   6. case*
   7. visit*
   8. admission*
   9. death*
   10. surveill*
   11. Fetal Mortality/ or Child Mortality/ or Infant Mortality/ or Hospital Mortality/ or Mortality/
   12. Mortalit*
   13. Diarrh*
   14. Diarrhea/ or Diarrhea, Infantile/
   15. 4 or 5 or 6 or 7 or 8 or 9 or 10 or 11 or 12 or 13 or 14
   16. 3 and 15
   17. limit 16 to (humans and yr="1990 -Current" and ("all infant (birth to 23 months)" or "all child (0 to 18 years)" or "newborn infant (birth to 1 month)" or "infant (1 to 23 months)" or "preschool child (2 to 5 years)") and (english or french or polish or spanish))
2. **Limits:**

- English, French, Spanish, Polish,
- Humans
- Year 1990- current
- Yr 0-5

1. **Search engines** (with modified search terms where appropriate):

- EMBASE
- Cochrane
- Medline
- Chinese citation database

1. **Exclusion Criteria**

| - Rotavirus Not Reported |
| --- |
| - Age Information Not Reported |
| - No Cases <5 Years |
| - EIA/ELISA or PCR Not Used or Diagnostic Not Specified |
| - Hospital Acquired (Nosocomial) Infection |
| - Comorbidities (Other Medical Conditions) in Cases |
| - Systematic Review/Meta-Analysis |
| - In vitro or animal study |
| - Full-Text Not Found |
|  |

**Risk of bias assessment tool**

When reviewing studies, we expanded the exclusion criteria to exclude highly biased studies (see Methods: Literature Review). Additionally, following a consultation with Cochrane we designed a custom tool to assess the risk of bias in the remaining observational studies. We checked each study for the following criteria:

1. whether participants were representative of the general population;
2. whether participants’ characteristics were reported;
3. whether surveillance methods and outcomes were the same for all participants;
4. whether study assessors were blinded at outcome assessment; and,
5. whether drop-outs, withdrawals and missing data were described?

Each answer was given a score: 0 for “No”, 1 for “Unclear”, and 2 for “Yes”. The total score was calculated and studies with a score of 0-1 were considered to have a very low risk of bias; 2-4 low risk of bias; and 5-7 medium risk of bias (Appendix Table S1).

**Appendix Table S1: Assessment criteria and scores for risk of bias in studies with rotavirus disease age distributions in children aged <5 years**

|  | **Selection Bias** | **Baseline Confounding** | **Outcome Measurement** | **Blinding at Outcome Assessment** | **Description of Missing** |  |  |  |  |  |
| --- | --- | --- | --- | --- | --- | --- | --- | --- | --- | --- |
| **Ref** | **Are participants representative of the general population?** | **Are characteristics of participants reported?** | **Are surveillance and measurement of outcomes the same for all participants?** | **Are study assessors blinded at outcome assessment?** | **Are drop-outs, withdrawals and missing data described?** | **Total "Y"** | **Total "U"** | **Total "N"** | **Score** | **Risk of Bias** |
| (1) | Y | Y | Y | U | Y | 4 | 1 | 0 | 1 | VERY LOW |
| (2) | Y | Y | Y | U | Y | 4 | 1 | 0 | 1 | VERY LOW |
| (3) | Y | Y | N | Y | N | 3 | 0 | 2 | 4 | LOW |
| (4) | Y | Y | Y | U | Y | 4 | 1 | 0 | 1 | VERY LOW |
| (5) | Y | Y | Y | U | Y | 4 | 1 | 0 | 1 | VERY LOW |
| (6) | Y | Y | Y | Y | N | 4 | 0 | 1 | 2 | LOW |
| (7) | Y | N | Y | U | N | 2 | 1 | 2 | 5 | MEDIUM |
| (8) | Y | Y | Y | U | Y | 4 | 1 | 0 | 1 | VERY LOW |
| (9) | Y | Y | Y | U | Y | 4 | 1 | 0 | 1 | VERY LOW |
| (10) | Y | N | Y | U | N | 2 | 1 | 2 | 5 | MEDIUM |
| (11) | Y | N | Y | U | N | 2 | 1 | 2 | 5 | MEDIUM |
| (12) | Y | Y | Y | U | Y | 4 | 1 | 0 | 1 | VERY LOW |
| (13) | Y | Y | Y | U | Y | 4 | 1 | 0 | 1 | VERY LOW |
| (14) | Y | Y | Y | U | N | 3 | 1 | 1 | 3 | LOW |
| (15) | Y | Y | Y | U | N | 3 | 1 | 1 | 3 | LOW |
| (16) | Y | Y | Y | U | Y | 4 | 1 | 0 | 1 | VERY LOW |
| (17) | Y | N | Y | U | N | 2 | 1 | 2 | 5 | MEDIUM |
| (18) | Y | N | Y | U | N | 2 | 1 | 2 | 5 | MEDIUM |
| (19) | Y | Y | Y | U | Y | 4 | 1 | 0 | 1 | VERY LOW |
| (20) | Y | Y | Y | Y | Y | 5 | 0 | 0 | 0 | VERY LOW |
| (21) | Y | Y | Y | U | Y | 4 | 1 | 0 | 1 | VERY LOW |
| (22) | Y | Y | Y | U | N | 3 | 1 | 1 | 3 | LOW |
| (23) | Y | Y | Y | U | N | 3 | 1 | 1 | 3 | LOW |
| (24) | Y | Y | Y | U | Y | 4 | 1 | 0 | 1 | VERY LOW |
| (25) | Y | Y | Y | U | Y | 4 | 1 | 0 | 1 | VERY LOW |
| (26) | Y | Y | Y | U | Y | 4 | 1 | 0 | 1 | VERY LOW |
| (27) | Y | N | Y | U | Y | 3 | 1 | 1 | 3 | LOW |
| (28) | Y | Y | N | U | Y | 3 | 1 | 1 | 3 | LOW |
| (29) | Y | N | Y | U | N | 2 | 1 | 2 | 5 | MEDIUM |
| (30) | Y | Y | Y | U | Y | 4 | 1 | 0 | 1 | VERY LOW |
| (31) | Y | N | Y | U | N | 2 | 1 | 2 | 5 | MEDIUM |
| (32) | Y | Y | Y | U | N | 3 | 1 | 1 | 3 | LOW |
| (33) | Y | Y | Y | U | N | 3 | 1 | 1 | 3 | LOW |
| (34) | Y | Y | Y | U | N | 3 | 1 | 1 | 3 | LOW |
| (35) | Y | Y | Y | U | Y | 4 | 1 | 0 | 1 | VERY LOW |
| (36) | Y | Y | Y | U | Y | 4 | 1 | 0 | 1 | VERY LOW |
| (37) | Y | Y | Y | U | Y | 4 | 1 | 0 | 1 | VERY LOW |
| (38) | Y | N | Y | U | N | 2 | 1 | 2 | 5 | MEDIUM |
| (39) | Y | Y | Y | U | Y | 4 | 1 | 0 | 1 | VERY LOW |
| (40) | Y | N | Y | U | N | 2 | 1 | 2 | 5 | MEDIUM |
| (41) | Y | N | Y | U | N | 2 | 1 | 2 | 5 | MEDIUM |
| (42) | Y | Y | Y | U | Y | 4 | 1 | 0 | 1 | VERY LOW |
| (43) | Y | Y | Y | U | Y | 4 | 1 | 0 | 1 | VERY LOW |
| (44) | Y | Y | Y | U | N | 3 | 1 | 1 | 3 | LOW |
| (45) | Y | N | N | U | N | 1 | 1 | 3 | 7 | MEDIUM |
| (46) | Y | Y | Y | U | Y | 4 | 1 | 0 | 1 | VERY LOW |
| (47) | Y | Y | Y | U | Y | 4 | 1 | 0 | 1 | VERY LOW |

**Justification for choosing the Log Logistic age distribution**

We fitted a range of parametric distributions to all cases identified in the GRSN that were reported by day of age. This dataset was used to identify the best-fitting parametric distributions because it had finest age granularity and covered a wide range of countries. Importantly, it also included a country dataset very skewed to younger ages (Zambia) and a country dataset very skewed to older ages (Ukraine) and represented the full range of age distributions identified in our search. We fitted separate parametric distributions to the aggregated global dataset, each WHO region, Zambia and the Ukraine. The *Log Logistic* distribution had the lowest (most favourable) goodness of fit statistics (Kolmogorov-Smirnov, Cramer-von Mises, Anderson-Darling) for Zambia, WHO regions AFR, SEAR, WPR and the global dataset (Appendix Figures S1 & S2). The *Lognormal* distribution had marginally better goodness of fit statistics than *Log Logistic* in the Ukraine. The *Burr* distribution had marginally better goodness of fit criteria than *Log Logistic* for most datasets (Akaike's Information Criterion – AIC, Bayesian Information Criterion - BIC) but had much worse goodness of fit statistics. In the interests of using a flexible, defensible and standardised fitting approach, we used the *Log Logistic* distribution to fit curves to all datasets.

The Log Logistic age distribution is defined by a scale (α) and a shape (β) parameter. The scale parameter is conveniently the same as the median age. The proportion of rotavirus disease within each week of age is calculated by the following equation, where $x$ represents the age at the beginning of each week of age

$$\frac{{(\beta/\alpha)(x/\alpha)}^{\beta-1}}{{{(1 +(x/\alpha)}^{\beta})}^{2}}$$

**Appendix Figure S1: Age distribution of rotavirus-positive hospital admissions aged <5 years for all countries included in the WHO GRSN database with data reported by day of age, before the introduction of rotavirus vaccination: comparison of alternative fitted parametric distributions**


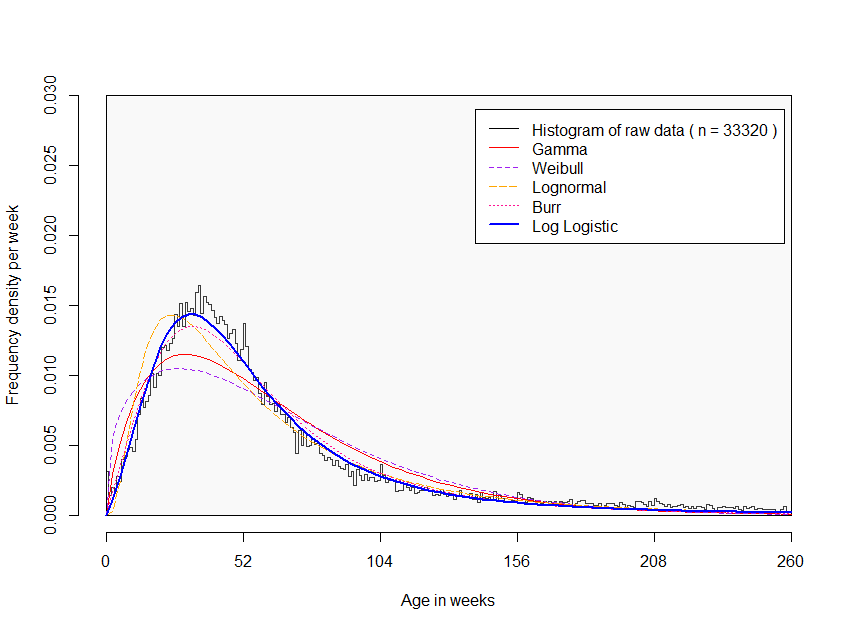


**Appendix Figure S2: Age distribution of rotavirus-positive hospital admissions aged <5 years for selected countries and WHO regions included in the WHO GRSN database with data reported by day of age, before the introduction of rotavirus vaccination: comparison of fitted parametric distributions**


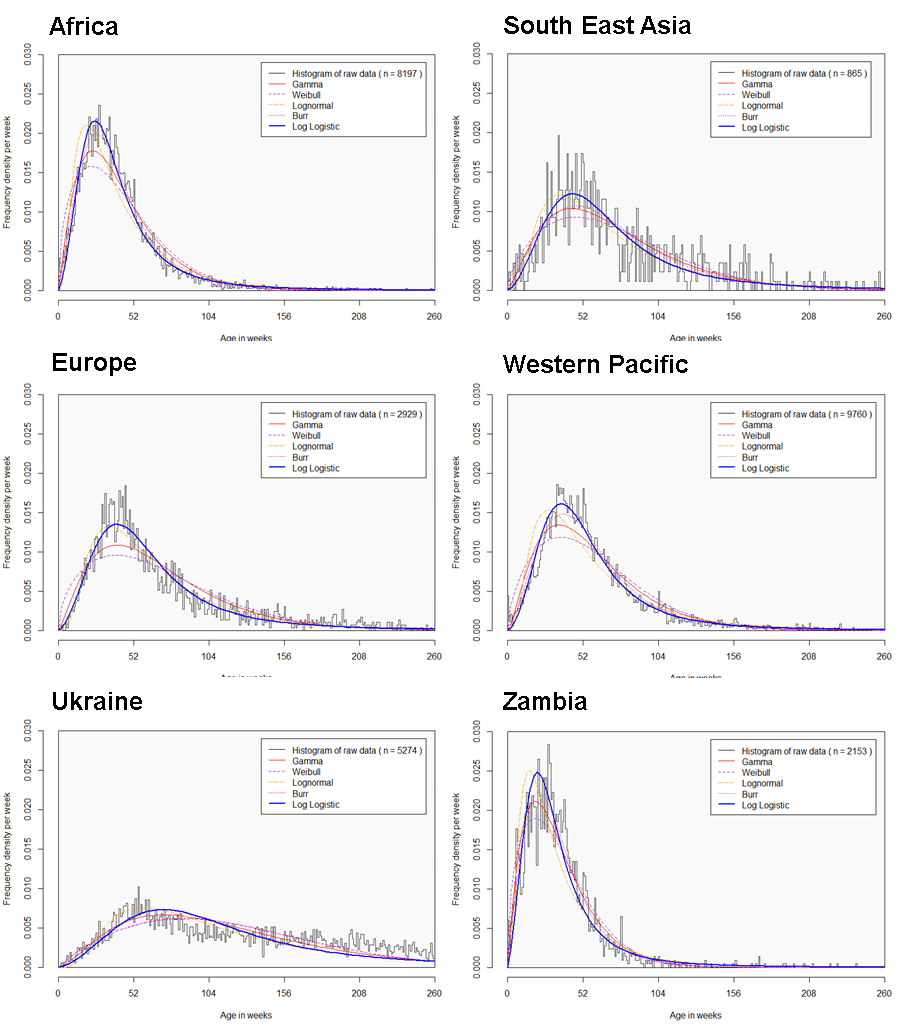


**Appendix Table S2. Age distributions for rotavirus-positive hospital admissions aged <5 years before the introduction of rotavirus vaccination: median, interquartile range and cumulative age distribution**

| **Study characteristics** | | | **Median (IQR)** | | | **Proportion of RVGE hospital admissions expected to occur by specific ages** | | | | | | | | | | | | |
| --- | --- | --- | --- | --- | --- | --- | --- | --- | --- | --- | --- | --- | --- | --- | --- | --- | --- | --- |
| **Ref** | **Country** | **n** | **50th pc.** | **25th pc.** | **75th pc.** | **6w** | **2m** | **10w** | **14w** | **15w** | **4m** | **6m** | **9m** | **12m** | **24m** | **36m** | **48m** | **60m** |
| (27) | Australia | **44** | **67** | 42 | 108 | 0·4% | 0·9% | 1·2% | 2·6% | 3·1% | 4·2% | 10·1% | 22·3% | 35·9% | 73·5% | 87·6% | 93·2% | 95·8% |
| (43) | Bulgaria | **2197** | **65** | 40 | 105 | 0·5% | 1·0% | 1·4% | 3·0% | 3·5% | 4·8% | 11·3% | 24·1% | 37·9% | 74·6% | 88·1% | 93·4% | 95·9% |
| (38) | Chile | **942** | **49** | 29 | 81 | 1·0% | 2·3% | 3·1% | 6·2% | 7·2% | 9·6% | 20·3% | 38·0% | 53·4% | 83·8% | 92·6% | 95·9% | 97·4% |
| (8) | China, Hong Kong SAR | **13147** | **69** | 41 | 116 | 0·5% | 1·2% | 1·6% | 3·2% | 3·6% | 4·9% | 10·9% | 22·6% | 35·1% | 70·5% | 85·0% | 91·3% | 94·4% |
| (28) | China, Hong Kong SAR | **123** | **84** | 53 | 133 | 0·2% | 0·4% | 0·6% | 1·3% | 1·6% | 2·2% | 5·6% | 13·6% | 23·9% | 62·4% | 81·5% | 89·8% | 93·7% |
| (3) | France | **8035** | **43** | 23 | 80 | 3·0% | 5·6% | 7·1% | 12·2% | 13·6% | 16·9% | 29·4% | 46·0% | 58·6% | 82·8% | 90·8% | 94·2% | 96·0% |
| (26) | France | **473** | **27** | 15 | 49 | 6·1% | 11·3% | 14·2% | 23·5% | 25·9% | 31·2% | 48·9% | 66·8% | 77·3% | 92·4% | 96·2% | 97·7% | 98·5% |
| (47) | Georgia | **110** | **67** | 42 | 107 | 0·4% | 0·9% | 1·2% | 2·6% | 3·0% | 4·2% | 10·1% | 22·3% | 35·9% | 73·8% | 87·8% | 93·4% | 95·9% |
| (3) | Hungary | **1820** | **86** | 52 | 142 | 0·3% | 0·7% | 0·9% | 1·8% | 2·1% | 2·9% | 6·8% | 15·1% | 25·0% | 60·4% | 78·8% | 87·5% | 91·9% |
| (7) | Italy | **1002** | **77** | 46 | 128 | 0·4% | 0·9% | 1·2% | 2·5% | 2·9% | 3·9% | 8·8% | 18·8% | 30·1% | 65·7% | 82·1% | 89·5% | 93·3% |
| (45) | Italy | **2434** | **62** | 33 | 119 | 1·9% | 3·4% | 4·3% | 7·4% | 8·2% | 10·3% | 18·6% | 31·2% | 42·5% | 70·6% | 82·7% | 88·6% | 91·9% |
| (23) | Japan | **58** | **73** | 48 | 113 | 0·2% | 0·4% | 0·6% | 1·5% | 1·7% | 2·5% | 6·7% | 16·7% | 29·4% | 70·8% | 87·2% | 93·4% | 96·1% |
| (3) | Netherlands | **710** | **48** | 25 | 92 | 3·0% | 5·4% | 6·8% | 11·3% | 12·6% | 15·5% | 26·5% | 41·6% | 53·6% | 78·7% | 87·9% | 92·2% | 94·5% |
| (25) | New Zealand | **555** | **55** | 35 | 87 | 0·4% | 1·1% | 1·5% | 3·4% | 4·0% | 5·5% | 13·6% | 29·7% | 46·1% | 82·2% | 92·6% | 96·2% | 97·7% |
| (47) | Oman | **728** | **53** | 35 | 78 | 0·3% | 0·7% | 1·0% | 2·5% | 3·1% | 4·5% | 12·6% | 30·5% | 49·2% | 86·7% | 95·2% | 97·8% | 98·8% |
| (40) | Russian Federation | **819** | **58** | 36 | 95 | 0·6% | 1·4% | 1·9% | 3·9% | 4·5% | 6·1% | 14·0% | 28·8% | 43·6% | 78·7% | 90·2% | 94·6% | 96·7% |
| (47) | Seychelles | **123** | **62** | 38 | 102 | 0·6% | 1·3% | 1·8% | 3·7% | 4·3% | 5·8% | 13·0% | 26·6% | 40·5% | 75·7% | 88·3% | 93·4% | 95·9% |
| (3) | Slovenia | **2224** | **88** | 58 | 134 | 0·1% | 0·2% | 0·3% | 0·8% | 0·9% | 1·3% | 3·8% | 10·4% | 19·8% | 60·6% | 81·8% | 90·6% | 94·5% |
| (47) | Sri Lanka | **153** | **65** | 46 | 93 | 0·1% | 0·2% | 0·3% | 0·8% | 1·0% | 1·6% | 5·4% | 16·8% | 32·9% | 80·8% | 93·7% | 97·3% | 98·6% |
| (22) | Switzerland | **586** | **64** | 38 | 107 | 0·6% | 1·4% | 1·8% | 3·7% | 4·3% | 5·7% | 12·6% | 25·6% | 39·0% | 73·8% | 87·1% | 92·6% | 95·3% |
| (47) | Ukraine | **5276** | **101** | 65 | 157 | 0·1% | 0·2% | 0·3% | 0·7% | 0·8% | 1·2% | 3·2% | 8·3% | 15·8% | 51·7% | 74·8% | 86·0% | 91·5% |
| **Very low / low mortality (median)** | | **728** | **65** | 40 | 107 | 0·4% | 1·0% | 1·4% | 3·0% | 3·5% | 4·8% | 11·3% | 24·1% | 37·9% | 73·8% | 87·8% | 93·4% | 95·9% |
| (47) | Armenia | **781** | **76** | 52 | 113 | 0·1% | 0·2% | 0·3% | 0·9% | 1·0% | 1·5% | 4·7% | 13·2% | 25·4% | 70·4% | 88·1% | 94·3% | 96·9% |
| (47) | China | **1078** | **52** | 33 | 81 | 0·5% | 1·2% | 1·7% | 3·9% | 4·6% | 6·4% | 15·6% | 33·4% | 50·4% | 84·8% | 93·8% | 96·8% | 98·2% |
| (47) | Egypt | **666** | **34** | 22 | 53 | 1·1% | 2·8% | 4·0% | 9·0% | 10·6% | 14·7% | 32·8% | 58·1% | 74·4% | 94·5% | 98·0% | 99·0% | 99·5% |
| (32) | Iran | **275** | **47** | 30 | 74 | 0·7% | 1·6% | 2·3% | 5·0% | 5·9% | 8·1% | 19·1% | 38·6% | 55·8% | 87·1% | 94·8% | 97·3% | 98·4% |
| (33) | Iran | **88** | **50** | 28 | 89 | 1·6% | 3·2% | 4·2% | 7·8% | 8·8% | 11·3% | 21·8% | 38·0% | 51·7% | 80·4% | 90·0% | 94·0% | 96·0% |
| (47) | Iran | **958** | **46** | 29 | 72 | 0·7% | 1·7% | 2·4% | 5·3% | 6·2% | 8·6% | 20·1% | 40·2% | 57·5% | 87·9% | 95·1% | 97·5% | 98·5% |
| (47) | Jordan | **337** | **32** | 21 | 50 | 1·6% | 3·8% | 5·3% | 11·4% | 13·2% | 17·9% | 37·1% | 61·4% | 76·3% | 94·7% | 98·0% | 99·0% | 99·4% |
| (14) | Kazakhstan | **2023** | **46** | 29 | 72 | 0·7% | 1·6% | 2·3% | 5·1% | 6·0% | 8·3% | 19·7% | 39·8% | 57·2% | 87·9% | 95·1% | 97·5% | 98·6% |
| (47) | Mauritius | **355** | **84** | 54 | 132 | 0·2% | 0·4% | 0·5% | 1·2% | 1·5% | 2·1% | 5·4% | 13·3% | 23·6% | 62·8% | 82·0% | 90·2% | 94·1% |
| (47) | Mongolia | **1424** | **38** | 26 | 55 | 0·4% | 1·3% | 1·9% | 5·1% | 6·1% | 9·1% | 24·7% | 52·0% | 71·6% | 95·1% | 98·5% | 99·3% | 99·7% |
| (47) | Syria | **1157** | **35** | 23 | 53 | 0·8% | 2·3% | 3·3% | 7·8% | 9·2% | 13·0% | 30·8% | 57·1% | 74·3% | 94·9% | 98·2% | 99·2% | 99·5% |
| (47) | Tunisia | **118** | **40** | 26 | 61 | 0·8% | 2·0% | 2·9% | 6·5% | 7·6% | 10·7% | 25·2% | 48·7% | 66·4% | 92·1% | 97·0% | 98·6% | 99·2% |
| (13) | Venezuela | **102** | **42** | 27 | 66 | 0·9% | 2·2% | 3·0% | 6·6% | 7·7% | 10·5% | 23·9% | 45·5% | 62·5% | 89·9% | 95·9% | 97·9% | 98·8% |
| (47) | Vietnam | **4628** | **52** | 35 | 76 | 0·2% | 0·6% | 0·9% | 2·3% | 2·8% | 4·1% | 12·2% | 30·7% | 50·3% | 88·1% | 95·9% | 98·2% | 99·0% |
| **Medium mortality (median)** | | **724** | **46** | 29 | 72 | 0·7% | 1·7% | 2·4% | 5·2% | 6·2% | 8·8% | 21·0% | 40·0% | 57·3% | 88·0% | 95·5% | 97·7% | 98·7% |
| (47) | Azerbaijan | **643** | **71** | 45 | 113 | 0·3% | 0·7% | 0·9% | 2·1% | 2·4% | 3·4% | 8·4% | 19·4% | 32·3% | 71·2% | 86·6% | 92·8% | 95·6% |
| (2) | Bangladesh | **297** | **35** | 19 | 65 | 3·8% | 7·2% | 9·2% | 15·7% | 17·4% | 21·5% | 36·4% | 54·5% | 66·9% | 87·7% | 93·7% | 96·2% | 97·4% |
| (10) | Bangladesh | **191** | **53** | 39 | 73 | 0·0% | 0·2% | 0·3% | 0·9% | 1·2% | 1·9% | 7·5% | 25·1% | 47·8% | 91·2% | 97·7% | 99·2% | 99·6% |
| (19) | Bangladesh | **2021** | **45** | 33 | 61 | 0·1% | 0·3% | 0·5% | 1·5% | 1·9% | 3·2% | 12·3% | 37·4% | 62·5% | 95·2% | 98·8% | 99·6% | 99·8% |
| (42) | Bangladesh | **4787** | **48** | 36 | 63 | 0·0% | 0·1% | 0·2% | 0·8% | 1·0% | 1·8% | 8·4% | 31·2% | 58·5% | 95·6% | 99·1% | 99·7% | 99·9% |
| (47) | Cambodia | **2094** | **48** | 33 | 70 | 0·2% | 0·6% | 0·9% | 2·4% | 2·9% | 4·4% | 13·5% | 34·5% | 55·5% | 90·9% | 97·1% | 98·8% | 99·4% |
| (47) | Eritrea | **110** | **67** | 42 | 107 | 0·4% | 0·9% | 1·2% | 2·6% | 3·0% | 4·2% | 10·1% | 22·3% | 35·9% | 73·8% | 87·9% | 93·4% | 96·0% |
| (10) | Guatemala | **91** | **47** | 28 | 78 | 1·2% | 2·7% | 3·6% | 7·1% | 8·2% | 10·8% | 22·3% | 40·4% | 55·6% | 84·5% | 92·8% | 96·0% | 97·5% |
| (10) | India | **173** | **52** | 38 | 70 | 0·0% | 0·1% | 0·2% | 0·8% | 1·0% | 1·8% | 7·4% | 26·2% | 50·6% | 92·9% | 98·3% | 99·4% | 99·7% |
| (20) | India | **704** | **43** | 27 | 69 | 1·0% | 2·3% | 3·1% | 6·6% | 7·7% | 10·5% | 23·2% | 43·9% | 60·6% | 88·7% | 95·3% | 97·5% | 98·5% |
| (30) | India | **52** | **54** | 38 | 78 | 0·1% | 0·4% | 0·6% | 1·6% | 2·0% | 3·0% | 9·7% | 27·0% | 47·0% | 88·0% | 96·2% | 98·4% | 99·2% |
| (34) | Indonesia | **88** | **48** | 32 | 73 | 0·4% | 1·1% | 1·6% | 3·8% | 4·5% | 6·4% | 16·6% | 36·5% | 54·9% | 88·2% | 95·6% | 97·9% | 98·8% |
| (47) | Indonesia | **711** | **60** | 39 | 94 | 0·3% | 0·8% | 1·1% | 2·5% | 3·0% | 4·2% | 10·8% | 25·0% | 40·7% | 79·6% | 91·5% | 95·7% | 97·5% |
| (47) | Iraq | **1786** | **32** | 19 | 52 | 2·3% | 5·2% | 7·0% | 13·8% | 15·7% | 20·5% | 39·1% | 61·5% | 75·3% | 93·6% | 97·3% | 98·6% | 99·1% |
| (29) | Kenya | **429** | **41** | 28 | 60 | 0·5% | 1·3% | 1·9% | 4·7% | 5·7% | 8·3% | 22·2% | 47·1% | 66·7% | 93·4% | 97·8% | 99·0% | 99·5% |
| (31) | Kenya | **232** | **26** | 17 | 40 | 2·5% | 6·1% | 8·4% | 17·6% | 20·2% | 26·7% | 50·1% | 73·4% | 85·0% | 97·0% | 98·9% | 99·4% | 99·7% |
| (37) | Kenya | **195** | **33** | 22 | 49 | 0·9% | 2·4% | 3·5% | 8·4% | 10·0% | 14·2% | 33·7% | 60·9% | 77·6% | 95·9% | 98·6% | 99·4% | 99·7% |
| (39) | Kenya | **587** | **42** | 29 | 61 | 0·3% | 0·9% | 1·4% | 3·8% | 4·6% | 6·9% | 19·7% | 44·9% | 65·6% | 93·7% | 98·0% | 99·1% | 99·6% |
| (47) | Kenya | **634** | **39** | 25 | 63 | 1·2% | 2·8% | 3·8% | 8·1% | 9·4% | 12·7% | 27·3% | 49·3% | 65·6% | 90·6% | 96·2% | 98·0% | 98·8% |
| (47) | Lao | **482** | **56** | 39 | 81 | 0·1% | 0·4% | 0·6% | 1·5% | 1·9% | 2·8% | 9·0% | 24·9% | 43·9% | 86·2% | 95·4% | 98·0% | 99·0% |
| (47) | Libya | **717** | **38** | 24 | 58 | 1·0% | 2·4% | 3·4% | 7·6% | 8·9% | 12·3% | 28·1% | 52·1% | 69·2% | 92·8% | 97·3% | 98·7% | 99·2% |
| (47) | Madagascar | **58** | **38** | 26 | 55 | 0·4% | 1·3% | 2·0% | 5·1% | 6·2% | 9·1% | 25·0% | 52·3% | 71·9% | 95·2% | 98·5% | 99·3% | 99·7% |
| (11) | Morocco | **582** | **36** | 22 | 60 | 2·1% | 4·5% | 6·0% | 11·6% | 13·2% | 17·1% | 33·1% | 54·1% | 68·7% | 90·7% | 95·9% | 97·7% | 98·6% |
| (47) | Morocco | **450** | **43** | 26 | 73 | 1·6% | 3·3% | 4·5% | 8·6% | 9·9% | 12·9% | 25·7% | 44·8% | 59·8% | 86·4% | 93·7% | 96·5% | 97·8% |
| (47) | Myanmar | **919** | **50** | 35 | 70 | 0·1% | 0·4% | 0·6% | 1·7% | 2·2% | 3·4% | 11·3% | 31·8% | 53·9% | 91·5% | 97·5% | 99·0% | 99·5% |
| (47) | Nepal | **536** | **38** | 21 | 68 | 3·3% | 6·2% | 8·0% | 13·9% | 15·5% | 19·3% | 33·6% | 51·7% | 64·5% | 86·7% | 93·3% | 95·9% | 97·3% |
| (47) | Senegal | **228** | **42** | 23 | 75 | 2·8% | 5·3% | 6·8% | 11·9% | 13·3% | 16·7% | 29·7% | 47·1% | 60·2% | 84·5% | 92·0% | 95·1% | 96·7% |
| (41) | South Africa | **216** | **27** | 18 | 41 | 2·0% | 5·0% | 7·0% | 15·4% | 17·9% | 24·1% | 47·8% | 72·5% | 84·8% | 97·1% | 99·0% | 99·5% | 99·7% |
| (47) | Tajikistan | **1395** | **42** | 30 | 59 | 0·2% | 0·6% | 0·9% | 2·6% | 3·3% | 5·2% | 16·9% | 43·2% | 66·0% | 94·9% | 98·6% | 99·4% | 99·7% |
| (14) | Uzbekistan | **1081** | **50** | 32 | 77 | 0·5% | 1·2% | 1·8% | 4·0% | 4·7% | 6·6% | 16·4% | 35·1% | 52·7% | 86·3% | 94·6% | 97·3% | 98·4% |
| (47) | Yemen | **1068** | **37** | 25 | 57 | 0·8% | 2·2% | 3·1% | 7·1% | 8·4% | 11·8% | 27·8% | 52·6% | 70·1% | 93·5% | 97·6% | 98·9% | 99·4% |
| **High mortality (median)** | | **536** | **43** | 28 | 68 | 0·5% | 1·3% | 1·9% | 4·7% | 5·7% | 8·3% | 22·2% | 43·9% | 60·6% | 90·9% | 97·1% | 98·6% | 99·2% |
| (47) | Afghanistan | **4628** | **36** | 24 | 55 | 0·9% | 2·4% | 3·4% | 7·9% | 9·3% | 12·9% | 29·8% | 54·8% | 71·9% | 93·9% | 97·8% | 98·9% | 99·4% |
| (47) | Benin | **125** | **37** | 28 | 50 | 0·1% | 0·5% | 0·8% | 2·7% | 3·5% | 5·7% | 21·2% | 54·3% | 77·3% | 97·7% | 99·5% | 99·8% | 99·9% |
| (47) | Cameroon | **1263** | **31** | 20 | 48 | 1·8% | 4·2% | 5·9% | 12·6% | 14·6% | 19·5% | 39·7% | 64·0% | 78·3% | 95·2% | 98·2% | 99·1% | 99·5% |
| (47) | Central African Republic | **293** | **31** | 21 | 44 | 0·7% | 2·2% | 3·4% | 8·7% | 10·5% | 15·3% | 37·9% | 67·4% | 83·0% | 97·5% | 99·3% | 99·7% | 99·8% |
| (47) | Côte d'Ivoire | **82** | **37** | 26 | 53 | 0·4% | 1·2% | 1·9% | 5·0% | 6·1% | 9·1% | 25·6% | 54·0% | 73·7% | 95·8% | 98·7% | 99·5% | 99·7% |
| (5) | DR Congo | **272** | **36** | 26 | 51 | 0·3% | 1·0% | 1·5% | 4·4% | 5·4% | 8·4% | 25·3% | 55·5% | 75·9% | 96·7% | 99·1% | 99·6% | 99·8% |
| (47) | DR Congo | **1750** | **33** | 23 | 48 | 0·5% | 1·5% | 2·3% | 6·3% | 7·7% | 11·6% | 31·5% | 61·8% | 79·8% | 97·1% | 99·2% | 99·7% | 99·8% |
| (47) | Ethiopia | **518** | **50** | 30 | 81 | 0·9% | 2·1% | 2·8% | 5·7% | 6·6% | 8·9% | 19·3% | 37·0% | 52·6% | 83·7% | 92·7% | 96·0% | 97·5% |
| (10) | Gambia | **86** | **50** | 34 | 74 | 0·3% | 0·7% | 1·0% | 2·7% | 3·2% | 4·8% | 13·5% | 32·9% | 52·5% | 88·6% | 96·1% | 98·2% | 99·0% |
| (47) | Ghana | **1133** | **39** | 25 | 61 | 1·0% | 2·5% | 3·5% | 7·6% | 8·9% | 12·2% | 27·3% | 50·3% | 67·1% | 91·8% | 96·8% | 98·4% | 99·1% |
| (47) | Guinea-Bissau | **443** | **40** | 25 | 63 | 1·0% | 2·5% | 3·5% | 7·5% | 8·8% | 11·9% | 26·5% | 48·8% | 65·5% | 91·0% | 96·4% | 98·2% | 98·9% |
| (47) | Lesotho | **86** | **38** | 26 | 57 | 0·5% | 1·5% | 2·2% | 5·4% | 6·5% | 9·5% | 24·8% | 51·0% | 70·1% | 94·3% | 98·1% | 99·2% | 99·6% |
| (17) | Malawi | **807** | **31** | 21 | 48 | 1·3% | 3·2% | 4·7% | 10·6% | 12·4% | 17·2% | 37·7% | 63·8% | 79·0% | 95·9% | 98·6% | 99·3% | 99·6% |
| (35) | Nigeria | **49** | **40** | 26 | 62 | 0·8% | 2·0% | 2·8% | 6·4% | 7·5% | 10·5% | 24·7% | 47·8% | 65·5% | 91·7% | 96·9% | 98·5% | 99·1% |
| (47) | Nigeria | **1336** | **39** | 27 | 58 | 0·5% | 1·3% | 2·0% | 5·0% | 6·0% | 8·8% | 23·5% | 49·2% | 68·7% | 94·0% | 98·0% | 99·1% | 99·5% |
| (47) | Pakistan | **3276** | **37** | 21 | 63 | 2·4% | 5·0% | 6·6% | 12·3% | 13·9% | 17·8% | 33·2% | 53·3% | 67·2% | 89·4% | 95·1% | 97·2% | 98·2% |
| (47) | Rwanda | **123** | **47** | 31 | 72 | 0·5% | 1·2% | 1·8% | 4·1% | 4·9% | 6·9% | 17·6% | 37·9% | 56·2% | 88·6% | 95·7% | 97·9% | 98·8% |
| (47) | Sierra Leone | **74** | **39** | 24 | 63 | 1·4% | 3·2% | 4·4% | 8·9% | 10·3% | 13·7% | 28·5% | 50·0% | 65·8% | 90·2% | 95·9% | 97·8% | 98·7% |
| (47) | Sudan | **4077** | **39** | 25 | 59 | 0·8% | 2·1% | 3·0% | 6·9% | 8·1% | 11·4% | 26·6% | 50·6% | 68·1% | 92·7% | 97·3% | 98·7% | 99·2% |
| (47) | Swaziland | **138** | **35** | 22 | 56 | 1·4% | 3·4% | 4·7% | 10·0% | 11·5% | 15·6% | 32·7% | 56·1% | 71·8% | 93·0% | 97·2% | 98·6% | 99·2% |
| (47) | Tanzania | **66** | **37** | 26 | 53 | 0·4% | 1·3% | 2·0% | 5·2% | 6·3% | 9·4% | 25·9% | 53·9% | 73·4% | 95·6% | 98·7% | 99·4% | 99·7% |
| (47) | Togo | **293** | **36** | 23 | 58 | 1·5% | 3·4% | 4·6% | 9·7% | 11·2% | 15·0% | 31·3% | 54·0% | 69·7% | 92·1% | 96·8% | 98·3% | 99·0% |
| (4) | Uganda | **263** | **41** | 29 | 58 | 0·2% | 0·6% | 1·0% | 2·9% | 3·6% | 5·7% | 18·4% | 45·6% | 68·0% | 95·3% | 98·7% | 99·5% | 99·7% |
| (47) | Uganda | **211** | **44** | 30 | 63 | 0·2% | 0·7% | 1·1% | 3·0% | 3·7% | 5·6% | 17·0% | 41·5% | 63·1% | 93·4% | 98·0% | 99·2% | 99·6% |
| (47) | Zambia | **2149** | **29** | 19 | 46 | 1·9% | 4·6% | 6·5% | 13·7% | 15·9% | 21·3% | 42·5% | 66·9% | 80·5% | 95·8% | 98·4% | 99·2% | 99·6% |
| (47) | Zimbabwe | **2601** | **39** | 25 | 60 | 0·9% | 2·2% | 3·2% | 7·1% | 8·4% | 11·6% | 26·7% | 50·2% | 67·5% | 92·2% | 97·1% | 98·5% | 99·2% |
| **Very high mortality (median)** | | **293** | **38** | 25 | 58 | 0·8% | 2·1% | 2·9% | 6·6% | 7·9% | 11·5% | 26·5% | 52·1% | 69·2% | 93·7% | 97·9% | 99·0% | 99·4% |

**Appendix Table S3. Goodness of fit statistics, goodness of fit criteria and best-fitting parameters of Log Logistic age distributions fitted to age distributions for rotavirus-positive hospital admissions <5 years in different countries**

| **Study characteristics** | | | **Period** | | **Shape** | | | **Scale** | | | **COR** | **Goodness of fit statistics and criteria** | | | | | | |
| --- | --- | --- | --- | --- | --- | --- | --- | --- | --- | --- | --- | --- | --- | --- | --- | --- | --- | --- |
| Ref | Country | **Age grps. (n)** | **From** | **To** | **Est.** | **L95** | **U95** | **Est.** | **L95** | **U95** |  | **RM**  **SE** | **MAE** | **KS** | **CV** | **AD** | **AIC** | **BIC** |
| **Very low / low mortality** | | |  |  |  |  |  |  |  |  |  |  |  |  |  |  |  |  |
| (27) | Australia | 31 | 1984 | 1985 | 2·31 | 1·71 | 2·91 | 66·87 | 52·71 | 80·99 | 0·0953 | 5% | 4% | 0·14 | 0·17 | 1·72 | 468 | 472 |
| (43) | Bulgaria | 44 | 2005 | 2011 | 2·27 | 2·19 | 2·35 | 64·64 | 62·56 | 66·71 | 0·0442 | 2% | 1% | 0·06 | 1·17 | 11·61 | 23250 | 23262 |
| (38) | Chile | 95 | 2007 | 2010 | 2·17 | 2·05 | 2·29 | 48·82 | 46·35 | 51·33 | 0·0464 | 3% | 2% | 0·05 | 0·50 | 4·13 | 9530 | 9539 |
| (8) | Hong Kong | 60 | 1997 | 2011 | 2·14 | 2·11 | 2·17 | 69·28 | 68·31 | 70·24 | 0·0428 | 2% | 2% | 0·06 | 4·89 | 66·91 | 142364 | 142379 |
| (28) | Hong Kong | 56 | 2014 | 2015 | 2·40 | 2·04 | 2·76 | 84·17 | 73·51 | 94·86 | 0·0419 | 2% | 2% | 0·07 | 0·05 | 0·86 | 1357 | 1362 |
| (3) | France | 100 | 2007 | 2016 | 1·77 | 1·73 | 1·80 | 42·74 | 41·82 | 43·66 | 0·0618 | 3% | 2% | 0·05 | 5·88 | 56·62 | 81992 | 82006 |
| (26) | France | 20 | 1997 | 2000 | 1·83 | 1·70 | 1·97 | 26·65 | 24·38 | 28·92 | 0·0253 | 3% | 3% | 0·06 | 0·25 | 2·33 | 4389 | 4397 |
| (47) | Georgia | 74 | 2012 | 2012 | 2·33 | 1·97 | 2·68 | 66·68 | 57·29 | 76·11 | 0·0326 | 5% | 3% | 0·08 | 0·10 | 0·73 | 1170 | 1176 |
| (3) | Hungary | 100 | 2006 | 2016 | 2·19 | 2·11 | 2·28 | 85·82 | 82·69 | 88·93 | 0·0570 | 3% | 2% | 0·08 | 1·41 | 15·58 | 20349 | 20360 |
| (7) | Italy | 98 | 2009 | 2016 | 2·16 | 2·05 | 2·27 | 76·90 | 73·09 | 80·72 | 0·0654 | 2% | 2% | 0·07 | 0·85 | 8·84 | 11013 | 11023 |
| (45) | Italy | 99 | 2013 | 2016 | 1·70 | 1·64 | 1·76 | 62·12 | 59·63 | 64·64 | 0·0928 | 4% | 3% | 0·08 | 3·97 | 40·34 | 26657 | 26668 |
| (23) | Japan | 32 | 2004 | 2006 | 2·54 | 2·00 | 3·08 | 73·43 | 60·36 | 86·47 | 0·0360 | 3% | 2% | 0·10 | 0·07 | 0·46 | 620 | 624 |
| (3) | Netherlands | 95 | 2007 | 2016 | 1·68 | 1·57 | 1·78 | 47·72 | 44·16 | 51·28 | 0·0756 | 4% | 3% | 0·07 | 0·92 | 10·73 | 7432 | 7441 |
| (25) | New Zealand | 57 | 1998 | 2000 | 2·44 | 2·27 | 2·61 | 55·47 | 52·20 | 58·74 | 0·0410 | 3% | 2% | 0·10 | 0·51 | 5·02 | 5631 | 5640 |
| (47) | Oman | 51 | 2008 | 2010 | 2·75 | 2·59 | 2·92 | 52·59 | 50·20 | 54·99 | -0·0222 | 5% | 3% | 0·10 | 0·64 | 3·46 | 7208 | 7218 |
| (40) | Russia | 98 | 2005 | 2007 | 2·25 | 2·12 | 2·38 | 58·26 | 55·20 | 61·33 | 0·0122 | 3% | 2% | 0·04 | 0·22 | 2·57 | 8540 | 8550 |
| (47) | Seychelles | 85 | 2013 | 2016 | 2·19 | 1·87 | 2·51 | 61·97 | 53·24 | 70·71 | 0·0123 | 3% | 2% | 0·06 | 0·06 | 0·45 | 1308 | 1314 |
| (3) | Slovenia | 99 | 2007 | 2016 | 2·64 | 2·55 | 2·73 | 88·33 | 85·94 | 90·72 | 0·0424 | 2% | 2% | 0·06 | 0·73 | 12·08 | 24287 | 24299 |
| (47) | Sri Lanka | 90 | 2014 | 2016 | 3·10 | 2·69 | 3·51 | 65·40 | 59·56 | 71·24 | -0·0358 | 4% | 3% | 0·08 | 0·18 | 1·08 | 1548 | 1554 |
| (22) | Switzerland | 96 | 2002 | 2006 | 2·14 | 2·00 | 2·29 | 64·09 | 59·95 | 68·26 | 0·0352 | 1% | 1% | 0·05 | 0·17 | 2·85 | 6260 | 6269 |
| (47) | Ukraine | 258 | 2012 | 2016 | 2·52 | 2·46 | 2·57 | 101·17 | 99·26 | 103·08 | 0·0538 | 4% | 3% | 0·09 | 6·84 | 55·95 | 59369 | 59382 |
| **Medium mortality** | | |  |  |  |  |  |  |  |  |  |  |  |  |  |  |  |  |
| (47) | Armenia | 193 | 2012 | 2012 | 2·80 | 2·64 | 2·97 | 76·32 | 72·98 | 79·66 | 0·0056 | 2% | 1% | 0·04 | 0·17 | 1·77 | 8244 | 8254 |
| (47) | China | 180 | 2014 | 2016 | 2·46 | 2·33 | 2·58 | 51·68 | 49·56 | 53·84 | 0·0446 | 3% | 2% | 0·05 | 0·75 | 6·82 | 10792 | 10801 |
| (47) | Egypt | 35 | 2008 | 2010 | 2·57 | 2·41 | 2·74 | 34·36 | 32·60 | 36·11 | 0·0227 | 1% | 1% | 0·06 | 0·37 | 2·42 | 6095 | 6104 |
| (32) | Iran | 33 | 2008 | 2010 | 2·42 | 2·18 | 2·66 | 47·21 | 43·25 | 51·15 | 0·0033 | 1% | 1% | 0·05 | 0·12 | 1·07 | 2729 | 2736 |
| (33) | Iran | 34 | 2008 | 2009 | 1·94 | 1·59 | 2·28 | 50·22 | 41·01 | 59·45 | 0·0349 | 2% | 2% | 0·10 | 0·11 | 0·93 | 919 | 924 |
| (47) | Iran | 47 | 2008 | 2010 | 2·42 | 2·29 | 2·55 | 45·92 | 43·85 | 47·98 | -0·0049 | 3% | 2% | 0·07 | 0·42 | 2·68 | 9466 | 9476 |
| (47) | Jordan | 34 | 2008 | 2012 | 2·45 | 2·23 | 2·67 | 32·26 | 29·84 | 34·69 | 0·0349 | 2% | 1% | 0·07 | 0·25 | 1·78 | 3068 | 3075 |
| (14) | Kazakhstan | 98 | 2007 | 2009 | 2·44 | 2·35 | 2·54 | 46·19 | 44·78 | 47·59 | 0·0344 | 2% | 1% | 0·05 | 1·21 | 9·24 | 19849 | 19860 |
| (47) | Mauritius | 174 | 2010 | 2014 | 2·44 | 2·24 | 2·66 | 84·02 | 77·80 | 90·25 | 0·0348 | 2% | 2% | 0·06 | 0·17 | 1·77 | 3896 | 3904 |
| (47) | Mongolia | 138 | 2013 | 2016 | 2·94 | 2·81 | 3·07 | 37·97 | 36·82 | 39·14 | 0·0433 | 2% | 1% | 0·05 | 0·79 | 6·10 | 12914 | 12925 |
| (47) | Syria | 32 | 2008 | 2013 | 2·70 | 2·56 | 2·83 | 35·09 | 33·79 | 36·38 | 0·0274 | 1% | 1% | 0·06 | 0·65 | 5·69 | 10512 | 10522 |
| (47) | Tunisia | 31 | 2008 | 2010 | 2·55 | 2·17 | 2·93 | 39·80 | 34·90 | 44·71 | -0·0177 | 3% | 2% | 0·08 | 0·09 | 0·53 | 1123 | 1129 |
| (13) | Venezuela | 57 | 2003 | 2003 | 2·41 | 2·02 | 2·80 | 42·05 | 36·18 | 47·94 | 0·0125 | 1% | 1% | 0·04 | 0·02 | 0·15 | 990 | 995 |
| (47) | Vietnam | 232 | 2012 | 2016 | 2·87 | 2·80 | 2·94 | 51·80 | 50·91 | 52·70 | 0·0091 | 1% | 1% | 0·02 | 0·30 | 4·50 | 45154 | 45167 |
| **High mortality** | | |  |  |  |  |  |  |  |  |  |  |  |  |  |  |  |  |
| (47) | Azerbaijan | 206 | 2012 | 2016 | 2·37 | 2·22 | 2·52 | 71·04 | 66·96 | 75·09 | 0·0249 | 2% | 2% | 0·05 | 0·30 | 2·72 | 6886 | 6895 |
| (2) | Bangladesh | 35 | 2012 | 2012 | 1·82 | 1·64 | 1·99 | 35·35 | 31·58 | 39·12 | 0·0406 | 7% | 4% | 0·12 | 0·57 | 3·95 | 2922 | 2929 |
| (10) | Bangladesh | 57 | 2007 | 2010 | 3·50 | 3·08 | 3·92 | 53·32 | 49·61 | 57·02 | -0·0004 | 1% | 1% | 0·05 | 0·06 | 0·40 | 1811 | 1817 |
| (19) | Bangladesh | 81 | 2000 | 2006 | 3·57 | 3·44 | 3·70 | 45·04 | 44·09 | 46·00 | 0·0103 | 1% | 1% | 0·05 | 0·67 | 3·80 | 18307 | 18318 |
| (42) | Bangladesh | 42 | 2012 | 2017 | 3·93 | 3·84 | 4·03 | 47·67 | 47·08 | 48·27 | 0·0207 | 1% | 1% | 0·07 | 2·64 | 16·03 | 42899 | 42912 |
| (47) | Cambodia | 178 | 2013 | 2016 | 3·00 | 2·89 | 3·10 | 48·31 | 47·11 | 49·50 | 0·0085 | 1% | 1% | 0·02 | 0·08 | 0·92 | 19968 | 19979 |
| (47) | Eritrea | 74 | 2013 | 2013 | 2·33 | 1·97 | 2·68 | 66·67 | 57·27 | 76·08 | 0·0333 | 5% | 3% | 0·08 | 0·10 | 0·74 | 1170 | 1175 |
| (10) | Guatemala | 46 | 2007 | 2009 | 2·13 | 1·76 | 2·49 | 46·81 | 38·99 | 54·65 | 0·0885 | 6% | 4% | 0·09 | 0·18 | 1·43 | 914 | 919 |
| (10) | India | 55 | 2007 | 2010 | 3·68 | 3·22 | 4·14 | 51·67 | 48·07 | 55·28 | -0·0167 | 1% | 1% | 0·05 | 0·04 | 0·29 | 1615 | 1621 |
| (20) | India | 19 | 2005 | 2007 | 2·34 | 2·20 | 2·49 | 43·28 | 40·95 | 45·60 | 0·0438 | 3% | 2% | 0·10 | 0·94 | 7·14 | 6866 | 6875 |
| (30) | India | 28 | 2011 | 2012 | 3·04 | 2·33 | 3·76 | 54·09 | 46·11 | 62·06 | 0·0161 | 4% | 3% | 0·09 | 0·12 | 0·96 | 511 | 515 |
| (34) | Indonesia | 27 | 2013 | 2013 | 2·61 | 2·16 | 3·07 | 48·22 | 41·52 | 54·91 | 0·0020 | 2% | 2% | 0·08 | 0·06 | 0·33 | 867 | 872 |
| (47) | Indonesia | 175 | 2014 | 2016 | 2·51 | 2·35 | 2·66 | 60·43 | 57·34 | 63·50 | 0·0117 | 2% | 1% | 0·03 | 0·09 | 1·36 | 7326 | 7335 |
| (47) | Iraq | 48 | 2008 | 2011 | 2·25 | 2·16 | 2·34 | 31·64 | 30·51 | 32·76 | 0·0001 | 2% | 2% | 0·06 | 0·80 | 5·31 | 16585 | 16596 |
| (29) | Kenya | 43 | 2009 | 2014 | 2·81 | 2·59 | 3·04 | 40·63 | 38·31 | 42·95 | -0·0089 | 3% | 2% | 0·08 | 0·36 | 2·46 | 4025 | 4033 |
| (31) | Kenya | 34 | 2009 | 2011 | 2·50 | 2·22 | 2·77 | 25·97 | 23·70 | 28·26 | -0·0601 | 3% | 2% | 0·13 | 0·63 | 3·74 | 2049 | 2056 |
| (37) | Kenya | 61 | 2005 | 2007 | 2·76 | 2·44 | 3·09 | 33·20 | 30·28 | 36·10 | 0·0060 | 3% | 2% | 0·07 | 0·08 | 0·51 | 1756 | 1763 |
| (39) | Kenya | 77 | 2002 | 2004 | 2·96 | 2·76 | 3·16 | 41·81 | 39·84 | 43·79 | 0·0262 | 1% | 1% | 0·05 | 0·21 | 1·27 | 5439 | 5448 |
| (47) | Kenya | 148 | 2008 | 2013 | 2·34 | 2·19 | 2·50 | 39·50 | 37·21 | 41·77 | -0·0082 | 1% | 1% | 0·03 | 0·10 | 0·76 | 6103 | 6111 |
| (47) | Lao | 137 | 2013 | 2016 | 2·99 | 2·77 | 3·21 | 56·44 | 53·49 | 59·40 | -0·0164 | 2% | 2% | 0·04 | 0·18 | 1·26 | 4755 | 4763 |
| (47) | Libya | 38 | 2008 | 2013 | 2·52 | 2·37 | 2·68 | 37·75 | 35·85 | 39·61 | 0·0120 | 2% | 1% | 0·07 | 0·36 | 2·43 | 6732 | 6741 |
| (47) | Madagascar | 38 | 2013 | 2013 | 2·95 | 2·30 | 3·60 | 37·78 | 32·23 | 43·33 | 0·0366 | 3% | 2% | 0·09 | 0·07 | 0·63 | 529 | 533 |
| (11) | Morocco | 34 | 2006 | 2009 | 2·15 | 2·00 | 2·29 | 36·10 | 33·73 | 38·45 | 0·0060 | 3% | 2% | 0·06 | 0·26 | 1·85 | 5592 | 5601 |
| (47) | Morocco | 38 | 2008 | 2009 | 2·10 | 1·94 | 2·26 | 43·07 | 39·76 | 46·36 | -0·0029 | 2% | 2% | 0·07 | 0·29 | 2·12 | 4502 | 4510 |
| (47) | Myanmar | 143 | 2014 | 2016 | 3·20 | 3·02 | 3·37 | 49·52 | 47·79 | 51·24 | 0·0160 | 1% | 1% | 0·03 | 0·10 | 0·84 | 8695 | 8705 |
| (47) | Nepal | 36 | 2014 | 2016 | 1·85 | 1·71 | 1·98 | 37·63 | 34·68 | 40·57 | 0·0517 | 4% | 3% | 0·08 | 0·50 | 5·21 | 5303 | 5311 |
| (47) | Senegal | 111 | 2009 | 2014 | 1·84 | 1·64 | 2·04 | 41·51 | 36·43 | 46·59 | 0·0638 | 4% | 3% | 0·08 | 0·24 | 2·10 | 2294 | 2301 |
| (41) | South Africa | 20 | 2003 | 2004 | 2·61 | 2·32 | 2·90 | 26·88 | 24·52 | 29·25 | 0·0076 | 4% | 3% | 0·08 | 0·18 | 1·07 | 1875 | 1882 |
| (47) | Tajikistan | 156 | 2012 | 2014 | 3·25 | 3·11 | 3·40 | 42·42 | 41·24 | 43·60 | 0·0055 | 1% | 1% | 0·03 | 0·25 | 1·41 | 12760 | 12770 |
| (14) | Uzbekistan | 92 | 2008 | 2009 | 2·50 | 2·38 | 2·63 | 49·82 | 47·78 | 51·87 | 0·0333 | 1% | 1% | 0·05 | 0·41 | 3·44 | 10716 | 10726 |
| (47) | Yemen | 38 | 2008 | 2011 | 2·61 | 2·47 | 2·74 | 37·48 | 36·00 | 38·96 | 0·0235 | 2% | 2% | 0·07 | 0·59 | 4·56 | 9919 | 9929 |
| **Very high mortality** | | |  |  |  |  |  |  |  |  |  |  |  |  |  |  |  |  |
| (47) | Afghanistan | 46 | 2008 | 2016 | 2·59 | 2·53 | 2·65 | 36·19 | 35·49 | 36·89 | 0·0095 | 3% | 2% | 0·08 | 3·12 | 18·70 | 42811 | 42823 |
| (47) | Benin | 56 | 2013 | 2016 | 3·66 | 3·11 | 4·22 | 37·21 | 34·20 | 40·21 | -0·0311 | 5% | 4% | 0·09 | 0·15 | 1·03 | 1094 | 1100 |
| (47) | Cameroon | 132 | 2008 | 2013 | 2·45 | 2·34 | 2·57 | 30·85 | 29·66 | 32·03 | 0·0290 | 2% | 1% | 0·04 | 0·42 | 4·04 | 11374 | 11385 |
| (47) | C. Afr. Rep. | 74 | 2011 | 2016 | 3·00 | 2·71 | 3·29 | 30·63 | 28·63 | 32·63 | 0·0187 | 1% | 1% | 0·05 | 0·08 | 0·72 | 2532 | 2539 |
| (47) | Côte d'Ivoire | 52 | 2010 | 2016 | 3·03 | 2·48 | 3·58 | 37·00 | 32·43 | 41·57 | 0·0446 | 3% | 2% | 0·07 | 0·10 | 0·57 | 739 | 744 |
| (5) | DR Congo | 24 | 2012 | 2013 | 3·22 | 2·90 | 3·54 | 36·39 | 34·09 | 38·71 | 0·0433 | 3% | 2% | 0·08 | 0·25 | 1·94 | 2395 | 2402 |
| (47) | DR Congo | 123 | 2009 | 2016 | 3·10 | 2·98 | 3·22 | 33·41 | 32·54 | 34·28 | 0·0246 | 1% | 1% | 0·03 | 0·30 | 3·00 | 15253 | 15264 |
| (47) | Ethiopia | 49 | 2008 | 2013 | 2·21 | 2·05 | 2·37 | 49·61 | 46·26 | 52·95 | 0·0064 | 2% | 2% | 0·04 | 0·12 | 1·31 | 5267 | 5275 |
| (10) | Gambia | 43 | 2007 | 2010 | 2·82 | 2·31 | 3·32 | 50·19 | 43·73 | 56·64 | 0·0419 | 4% | 3% | 0·07 | 0·06 | 0·54 | 838 | 843 |
| (47) | Ghana | 145 | 2009 | 2011 | 2·45 | 2·33 | 2·57 | 38·83 | 37·23 | 40·42 | 0·0363 | 2% | 1% | 0·04 | 0·28 | 3·18 | 10707 | 10717 |
| (47) | Guinea-Bis. | 120 | 2010 | 2013 | 2·40 | 2·22 | 2·59 | 39·79 | 37·12 | 42·47 | 0·0393 | 3% | 2% | 0·04 | 0·16 | 1·46 | 4223 | 4231 |
| (47) | Lesotho | 51 | 2013 | 2016 | 2·83 | 2·32 | 3·33 | 38·47 | 33·52 | 43·41 | 0·0420 | 2% | 2% | 0·06 | 0·06 | 0·43 | 793 | 798 |
| (17) | Malawi | 58 | 1997 | 2007 | 2·64 | 2·48 | 2·79 | 31·43 | 30·01 | 32·85 | 0·0320 | 2% | 1% | 0·08 | 0·42 | 4·28 | 7169 | 7178 |
| (35) | Nigeria | 19 | 2012 | 2013 | 2·54 | 1·95 | 3·13 | 40·36 | 32·60 | 48·12 | 0·0458 | 4% | 3% | 0·11 | 0·06 | 0·49 | 466 | 470 |
| (47) | Nigeria | 152 | 2010 | 2016 | 2·84 | 2·71 | 2·97 | 39·43 | 38·15 | 40·71 | 0·0143 | 1% | 1% | 0·03 | 0·21 | 1·57 | 12343 | 12354 |
| (47) | Pakistan | 53 | 2008 | 2016 | 2·05 | 1·99 | 2·10 | 36·59 | 35·53 | 37·66 | 0·0241 | 1% | 1% | 0·05 | 1·40 | 11·92 | 31740 | 31752 |
| (47) | Rwanda | 74 | 2010 | 2011 | 2·59 | 2·20 | 2·98 | 47·20 | 41·78 | 52·64 | 0·0439 | 3% | 2% | 0·06 | 0·13 | 1·52 | 1199 | 1205 |
| (47) | Sierra Leone | 26 | 2013 | 2013 | 2·27 | 1·82 | 2·71 | 38·99 | 32·40 | 45·58 | 0·0195 | 6% | 5% | 0·12 | 0·14 | 0·89 | 717 | 722 |
| (47) | Sudan | 47 | 2008 | 2010 | 2·56 | 2·50 | 2·63 | 38·65 | 37·86 | 39·45 | 0·0227 | 2% | 1% | 0·07 | 2·49 | 15·96 | 38282 | 38294 |
| (47) | Swaziland | 69 | 2013 | 2014 | 2·39 | 2·05 | 2·73 | 35·18 | 30·95 | 39·41 | 0·0606 | 3% | 2% | 0·10 | 0·24 | 1·46 | 1284 | 1290 |
| (47) | Tanzania | 45 | 2009 | 2012 | 2·98 | 2·37 | 3·60 | 37·01 | 31·96 | 42·06 | 0·0078 | 5% | 3% | 0·09 | 0·07 | 0·47 | 601 | 606 |
| (47) | Togo | 97 | 2009 | 2013 | 2·34 | 2·11 | 2·57 | 36·40 | 33·38 | 39·44 | 0·0341 | 2% | 2% | 0·05 | 0·13 | 1·57 | 2759 | 2766 |
| (4) | Uganda | 25 | 2012 | 2012 | 3·24 | 2·91 | 3·57 | 41·19 | 38·53 | 43·83 | 0·0263 | 3% | 2% | 0·07 | 0·18 | 1·33 | 2382 | 2389 |
| (47) | Uganda | 84 | 2015 | 2016 | 3·06 | 2·71 | 3·41 | 43·64 | 40·35 | 46·94 | 0·0290 | 3% | 2% | 0·05 | 0·10 | 0·90 | 1961 | 1968 |
| (47) | Zambia | 135 | 2008 | 2013 | 2·48 | 2·39 | 2·57 | 29·35 | 28·49 | 30·21 | 0·0490 | 3% | 2% | 0·05 | 1·13 | 10·37 | 19026 | 19038 |
| (47) | Zimbabwe | 190 | 2009 | 2013 | 2·51 | 2·43 | 2·60 | 38·86 | 37·84 | 39·88 | 0·0226 | 1% | 1% | 0·03 | 0·37 | 3·68 | 24521 | 24533 |

**COR**: Correlation coefficient between fitted shape and scale parameters

**Goodness of fit statistics and criteria**: RMSE = Root Mean Squared Error, MAE = Mean Absolute Error, KS = Kolmogorov-Smirnov statistic; CV= Cramer-von Mises statistic; AD= Anderson-Darling statistic; AIC = Akaike's Information Criterion; BIC = Bayesian Information Criterion.

**Regression models to predict scale and shape parameters of the Log Logistic distribution**

*Independent variables considered*

- 5 mortality strata (v low, low, medium, high, v high)
- 3 mortality strata (v low and low, medium, high and v high)
- 2 mortality strata (v low/ + low; medium + high + v high)
- WHO regions (6)
- WHO Choice mortality strata/subregions (14)
- Mid-year of data collection period (1980-1999, 2000-2004, 2005-2009, 2010-2014, 2015-2018).
- Presentation: clinic visits, community cases, emergency visits, hospital admissions.
- Access to skilled delivery: 0-35%, 36-44%, 45-54%, 55-64%, 65-94%, 95-100%.
- GDP per capita in 2011

*Datasets considered*

1. all 117 studies
2. 110 studies excluding 7 of emergency admissions
3. 106 studies excluding 7 of emergency admissions and 4 with midyear of data collection between 1980 and 1999.
4. 103 studies excluding 7 of emergency admissions, 4 with midyear of data collection between 1980 and 1999, and 3^[[1]](#footnote-1)^ with no data on GDP

**Appendix Table S4: Summary of alternative models for the Scale (median age) parameter**

| Model | Data | Independent variables | df | Akaike’s  Information  Criterion | Adjusted R^2^ (Scale) |
| --- | --- | --- | --- | --- | --- |
| 1 | A | 14 WHO Choice mortality sub-regions | 13 | 919.8 | 41.5% |
| 2 | A | 5 mortality strata | 4 | 920.7 | 36.8% |
| 3 | B | 5 mortality strata | 4 | 861.3 | 40.8% |
| 4 | C | 5 mortality strata | 4 | 815.9 | 47.6% |
| 5 | D | 5 mortality strata | 5 | 794.4 | 43.8% |
| 6 | D | 6 WHO regions and 2 mortality strata | 6 | 793.9 | 45.7% |
| 7 | D | 6 WHO regions and 3 mortality strata | 7 | 784.2 | 50.5% |
| 8 | D | 6 WHO regions and 5 mortality strata | 9 | 787.5 | 49.8% |
| 9 | D | 6 WHO regions and 3 mortality strata, with interactions | 15 | 777.0 | 56.9% |
| 10 | D | 5 mortality strata and GDPpc2011 | 5 | 784.4 | 49.5% |
| 11 | D | 6 WHO regions & 3 mortality strata with interactions, and GDPpc2011 | 16 | 765.4 | 61.8% |

By comparing models 2 to 5 in this table it can be seen that selecting less heterogeneous sets of studies for analysis resulted in better fits.

Using D, the most restricted dataset, it can then be seen that models 9 and 11 provide the best fit, in terms of both AIC and adjusted R-square. However they involve large numbers of predictors relative to the numbers of observations, including one for the mid strata and one for the high strata in each region, risking overfitting. For several combinations of stratum and region of the coefficient depends on only one data point, and for 2 combinations there is no data point.

Model 5, in which the predicted value of the scale parameter for a country is simply the mean of the scale parameters for all studies in that country’s mortality stratum, provides a moderately good fit with few predictors, and is worth considering for its simplicity.

Model 7 is superior to model 5 (likelihood ratio test p = 0.1%) and so is Model 10 (LR test p = 0.05%). There is no evidence that model 10 is superior to model 7 in terms of fit (LR test p = 12%) but model 10 involves fewer predictors.

For the Shape parameter, restricting the analysis to dataset D again improved the fit for the simple mortality strata model in terms of AIC (116.9 for model 5compared to 131.2 for model 2) but not in terms of adjusted R-squared (12.8% compared to 15.0%). However none of the models tried was good. For example model 7 gave R-square 16.9% and AIC 114.8.

**Appendix Table S5: Selected regression models for estimating the scale parameter of the Log Logistic age distribution**

|  |  | **Model 5** |  |  | **Model 7** |  |  | **Model 10** | |  |  |  |  |  | **Model 11** | | |
| --- | --- | --- | --- | --- | --- | --- | --- | --- | --- | --- | --- | --- | --- | --- | --- | --- | --- |
|  |  |  |  |  |  |  |  |  |  |  |  |  |  |  |  |  |  |
| R-Sq |  | 46.0% |  |  | 53.90% |  |  | 52.00% |  |  |  | R-Sq |  |  | 67.80% |  |  |
| adjusted R-sq | | 43.8% |  |  | 49.8% |  |  | 49.5% |  |  |  | adjusted R-sq | |  | 61.8% |  |  |
| AIC |  | 794.4 |  |  | 787.5 |  |  | 784.4 |  |  |  | AIC |  |  | 765.4 |  |  |
|  |  |  |  |  |  |  |  |  |  |  |  |  |  |  |  |  |  |
|  |  | *group n* | *coeff* | *p* | *group n* | *coeff* | *p* | *group n* | *coeff* | *p* |  |  |  |  | *group n* | *coeff* | *p* |
| Constant |  |  | 70.16 | 0.000 |  | 55.53 | 0.000 |  | 91.09 | 0.000 |  | Constant |  |  |  | 52.82 | 0.000 |
|  |  |  |  |  |  |  |  |  |  |  |  |  |  |  |  |  |  |
| GDP per capita | |  |  |  |  |  |  |  | -0.0006 | 0.001 |  | GDP |  |  |  | -0.0004 | 0.001 |
|  |  |  |  |  |  |  |  |  |  |  |  |  |  |  |  |  |  |
| mortality | Very low | 11 | 0.00 |  |  |  |  | 11 | 0.00 |  |  | region/ mortality | AFR | Low | 0 | - |  |
|  | Low | 8 | -6.22 | 0.234 | 19 | 0.00 |  | 8 | -21.93 | 0.001 |  |  |  | Mid | 1 | 34.76 | 0.003 |
|  | Mid | 15 | -22.12 | 0.000 | 15 | -9.58 | 0.032 | 15 | -40.15 | 0.000 |  |  |  | High | 36 | -13.23 | 0.052 |
|  | High | 38 | -25.36 | 0.000 | 69 | -15.31 | 0.000 | 38 | -45.17 | 0.000 |  |  | AMR | Low | 1 | 1.83 | 0.873 |
|  | Very high | 31 | -31.39 | 0.000 |  |  |  | 31 | -51.80 | 0.000 |  |  |  | Mid | 1 | -6.39 | 0.575 |
|  |  |  |  |  |  |  |  |  |  |  |  |  |  | High | 2 | -0.62 | 0.947 |
| region | AFR |  |  |  | 37 | 0.00 |  |  |  |  |  |  | EMR | Low | 1 | 9.97 | 0.394 |
|  | AMR |  |  |  | 4 | 2.69 | 0.638 |  |  |  |  |  |  | Mid | 7 | -10.50 | 0.160 |
|  | EMR |  |  |  | 18 | -3.92 | 0.240 |  |  |  |  |  |  | High | 10 | -14.58 | 0.044 |
|  | EUR |  |  |  | 19 | 14.99 | 0.001 |  |  |  |  |  | EUR | Low | 14 | 29.17 | 0.000 |
|  | SEAR |  |  |  | 18 | 6.53 | 0.035 |  |  |  |  |  |  | Mid | 2 | 11.38 | 0.222 |
|  | WPR |  |  |  | 7 | 6.50 | 0.183 |  |  |  |  |  |  | High | 3 | 2.87 | 0.734 |
|  |  |  |  |  |  |  |  |  |  |  |  |  | SEAR | Low | 2 | 8.45 | 0.362 |
|  |  |  |  |  |  |  |  |  |  |  |  |  |  | Mid | 0 | - |  |
|  |  |  |  |  |  |  |  |  |  |  |  |  |  | High | 16 | -5.22 | 0.452 |
|  |  |  |  |  |  |  |  |  |  |  |  |  | WPR | Low | 1 | 39.18 | 0.002 |
|  |  |  |  |  |  |  |  |  |  |  |  |  |  | Mid | 4 | -4.49 | 0.576 |
|  |  |  |  |  |  |  |  |  |  |  |  |  |  | High | 2 | 0.00 |  |

**Appendix Table S6: Age distributions for rotavirus-positive community cases aged <5 years before the introduction of rotavirus vaccination: median, interquartile range and cumulative age distribution***

| **Reference** | **Country** | **n** | **50th pc.** | **25th pc.** | **75th pc.** | **6w** | **2m** | **10w** | **14w** | **15w** | **4m** | **6m** | **9m** | **12m** | **24m** | **36m** | **48m** | **60m** |
| --- | --- | --- | --- | --- | --- | --- | --- | --- | --- | --- | --- | --- | --- | --- | --- | --- | --- | --- |
| (6) | Bangladesh | **143** | **44** | 31 | 61 | 0·2% | 0·5% | 0·8% | 2·4% | 2·9% | 4·7% | 15·6% | 41·1% | 64·1% | 94·6% | 98·5% | 99·4% | 99·7% |
| (6) | India | **40** | **43** | 29 | 65 | 0·5% | 1·3% | 2·0% | 4·7% | 5·6% | 8·0% | 20·5% | 43·3% | 62·3% | 91·4% | 96·9% | 98·5% | 99·2% |
| (44) | India | **282** | **37** | 19 | 70 | 4·4% | 7·9% | 9·8% | 16·2% | 17·9% | 21·7% | 35·6% | 52·4% | 64·2% | 85·4% | 92·1% | 95·0% | 96·5% |
| (46) | Mexico | **89** | **28** | 17 | 46 | 3·1% | 6·7% | 9·0% | 17·3% | 19·6% | 25·2% | 45·5% | 67·4% | 79·8% | 94·9% | 97·9% | 98·9% | 99·3% |
| (6) | Nepal | **71** | **50** | 35 | 70 | 0·1% | 0·4% | 0·6% | 1·8% | 2·3% | 3·6% | 11·6% | 32·0% | 53·8% | 91·1% | 97·4% | 98·9% | 99·5% |
| (6) | Pakistan | **71** | **37** | 25 | 56 | 0·7% | 2·0% | 2·8% | 6·7% | 8·0% | 11·3% | 27·4% | 52·7% | 70·7% | 93·9% | 97·9% | 99·0% | 99·4% |
|  | Median | **80** | **40** | 27 | 63 | 0·6% | 1·7% | 2·4% | 5·7% | 6·8% | 9·7% | 23·9% | 47·9% | 64·2% | 92·6% | 97·6% | 98·9% | 99·4% |

*These age distributions are likely to be biased to younger ages because all of the data used for the fitting was based on children aged <2 years.

**Appendix Table S7: Age distributions for rotavirus-positive clinic visits aged <5 years before the introduction of rotavirus vaccination: median, interquartile range and cumulative age distribution**

| **Reference** | **Country** | **n** | **50th pc.** | **25th pc.** | **75th pc.** | **6w** | **2m** | **10w** | **14w** | **15w** | **4m** | **6m** | **9m** | **12m** | **24m** | **36m** | **48m** | **60m** |
| --- | --- | --- | --- | --- | --- | --- | --- | --- | --- | --- | --- | --- | --- | --- | --- | --- | --- | --- |
| (36) | China | **224** | **46** | 29 | 72 | 0·7% | 1·8% | 2·5% | 5·4% | 6·4% | 8·8% | 20·4% | 40·6% | 57·8% | 88·0% | 95·1% | 97·5% | 98·5% |
| (9) | Gambia | **119** | **54** | 37 | 78 | 0·2% | 0·5% | 0·7% | 1·9% | 2·4% | 3·6% | 10·8% | 28·3% | 47·8% | 87·4% | 95·8% | 98·1% | 99·0% |
| (21) | Germany | **1244** | **58** | 33 | 101 | 1·1% | 2·2% | 2·9% | 5·5% | 6·3% | 8·2% | 16·8% | 31·2% | 44·6% | 76·2% | 87·8% | 92·7% | 95·2% |
| (10) | Guatemala | **56** | **55** | 36 | 84 | 0·3% | 0·8% | 1·2% | 2·8% | 3·4% | 4·8% | 12·6% | 29·1% | 46·4% | 83·8% | 93·6% | 96·9% | 98·2% |
| (3) | Hungary | **229** | **96** | 57 | 163 | 0·3% | 0·6% | 0·8% | 1·7% | 1·9% | 2·6% | 6·0% | 12·9% | 21·4% | 53·9% | 73·3% | 83·4% | 89·0% |
| (9) | India | **222** | **54** | 36 | 79 | 0·2% | 0·5% | 0·8% | 2·1% | 2·5% | 3·8% | 11·2% | 28·7% | 47·9% | 87·0% | 95·5% | 98·0% | 98·9% |
| (9) | Kenya | **183** | **40** | 26 | 61 | 0·8% | 2·0% | 2·8% | 6·4% | 7·6% | 10·6% | 25·2% | 48·8% | 66·6% | 92·2% | 97·1% | 98·6% | 99·2% |
| (17) | Malawi | **446** | **35** | 25 | 51 | 0·5% | 1·4% | 2·2% | 5·7% | 7·0% | 10·4% | 28·3% | 57·4% | 76·3% | 96·3% | 98·9% | 99·5% | 99·8% |
| (9) | Mali | **275** | **46** | 31 | 69 | 0·4% | 1·0% | 1·5% | 3·7% | 4·5% | 6·5% | 17·4% | 38·8% | 58·1% | 90·1% | 96·5% | 98·4% | 99·1% |
| (9) | Pakistan | **226** | **37** | 25 | 54 | 0·6% | 1·7% | 2·5% | 6·2% | 7·5% | 10·8% | 27·6% | 54·5% | 72·9% | 95·0% | 98·4% | 99·3% | 99·6% |
| (3) | Slovenia | **79** | **76** | 50 | 116 | 0·1% | 0·3% | 0·5% | 1·2% | 1·4% | 2·0% | 5·6% | 14·7% | 26·9% | 69·4% | 86·8% | 93·3% | 96·2% |
| (16) | Thailand | **41** | **54** | 33 | 88 | 0·7% | 1·7% | 2·3% | 4·7% | 5·4% | 7·3% | 16·4% | 32·6% | 47·9% | 81·2% | 91·5% | 95·3% | 97·1% |
|  | Median | **223** | **54** | 33 | 78 | 0·4% | 1·2% | 1·8% | 4·2% | 4·9% | 6·9% | 16·6% | 31·9% | 47·9% | 87·2% | 95·3% | 97·7% | 98·7% |

**Appendix Table S8: Age distributions for rotavirus-positive emergency visits aged <5 years before the introduction of rotavirus vaccination: median, interquartile range and cumulative age distribution**

| **Reference** | **Country** | **n** | **50th pc.** | **25th pc.** | **75th pc.** | **6w** | **2m** | **10w** | **14w** | **15w** | **4m** | **6m** | **9m** | **12m** | **24m** | **36m** | **48m** | **60m** |
| --- | --- | --- | --- | --- | --- | --- | --- | --- | --- | --- | --- | --- | --- | --- | --- | --- | --- | --- |
| (15) | France | **9127** | **41** | 21 | 78 | 3·7% | 6·6% | 8·3% | 13·9% | 15·3% | 18·8% | 31·6% | 48·0% | 60·1% | 83·1% | 90·7% | 94·1% | 95·9% |
| (1) | Israel | **1245** | **43** | 28 | 67 | 0·6% | 1·6% | 2·3% | 5·3% | 6·3% | 8·9% | 21·5% | 43·4% | 61·5% | 90·3% | 96·3% | 98·2% | 99·0% |
| (18) | Namibia | **119** | **33** | 18 | 61 | 4·4% | 8·2% | 10·4% | 17·5% | 19·4% | 23·8% | 39·2% | 57·2% | 69·1% | 88·6% | 94·1% | 96·4% | 97·6% |
| (3) | Netherlands | **95** | **58** | 36 | 92 | 0·5% | 1·2% | 1·7% | 3·6% | 4·2% | 5·7% | 13·5% | 28·7% | 44·0% | 79·8% | 91·1% | 95·2% | 97·1% |
| (41) | South Africa | **48** | **28** | 16 | 52 | 5·5% | 10·2% | 12·8% | 21·5% | 23·7% | 28·8% | 46·0% | 64·2% | 75·2% | 91·6% | 95·8% | 97·5% | 98·3% |
| (12) | USA | **101** | **63** | 41 | 98 | 0·3% | 0·7% | 1·0% | 2·3% | 2·7% | 3·9% | 9·9% | 23·2% | 38·1% | 77·5% | 90·4% | 95·1% | 97·1% |
| (24) | USA | **78** | **53** | 32 | 87 | 0·9% | 1·9% | 2·6% | 5·3% | 6·1% | 8·1% | 17·7% | 34·3% | 49·4% | 81·6% | 91·5% | 95·3% | 97·1% |
|  | Median | **101** | **43** | 28 | 78 | 0·9% | 1·9% | 2·6% | 5·3% | 6·3% | 8·9% | 21·5% | 43·4% | 60·1% | 83·1% | 91·5% | 95·3% | 97·1% |

**Appendix Figure S3: Median age of rotavirus disease before the introduction of rotavirus, by type of presentation and national under-five mortality rate**


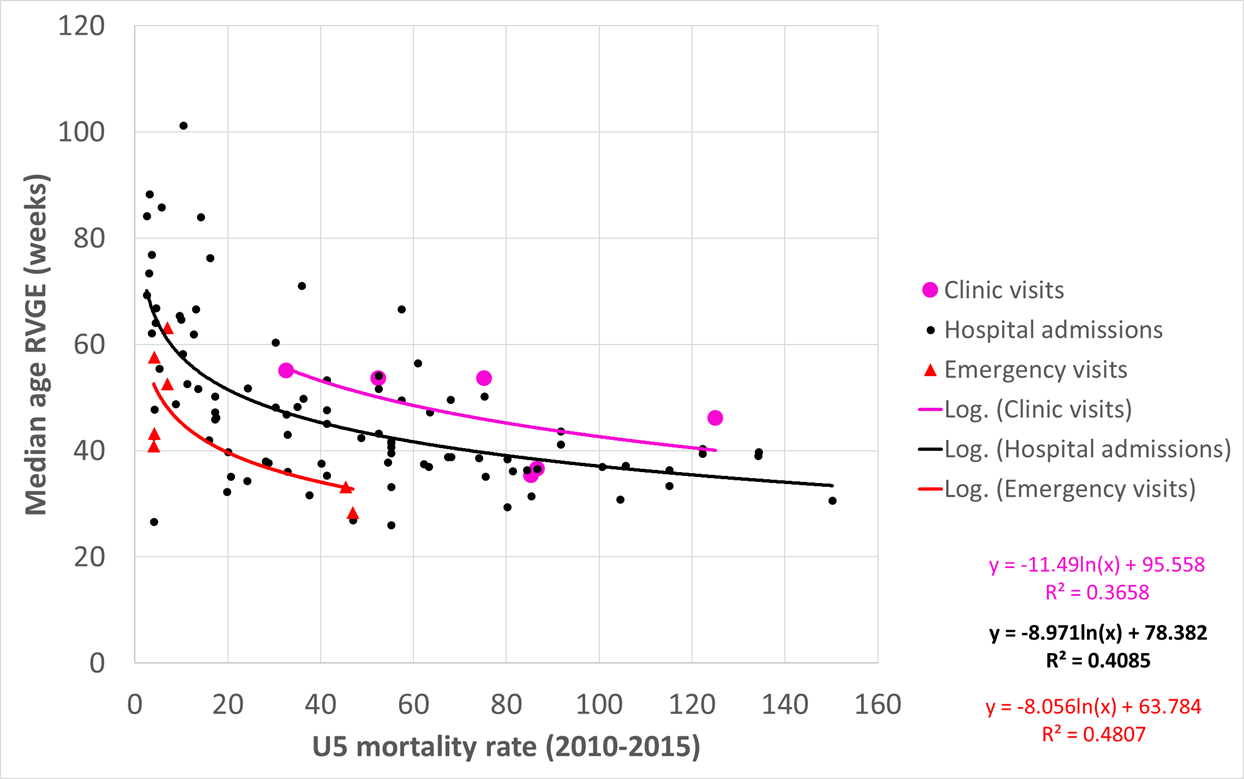


We did not include community cases in this analysis because the data were only available up to two years of age, compared to five years for other presentations.

**List of references**

1. Leshem E, Givon-Lavi N, Tate JE, Greenberg D, Parashar UD, Dagan R. Real-World Effectiveness of Pentavalent Rotavirus Vaccine among Bedouin and Jewish Children in Southern Israel. Clinical Infectious Diseases. 2016;62:S155-S60.

2. Arif MT, Asm NUA, Rajesh A, Belal H, Mahfuza M, Shampa S, et al. Rotavirus surveillance at a who-coordinated invasive bacterial disease surveillance site in Bangladesh: A feasibility study to integrate two surveillance systems. PLoS ONE. 2016;11.

3. Hungerford D, Vivancos R, Read JM, Pitzer VE, Cunliffe N, French N, et al. In-season and out-of-season variation of rotavirus genotype distribution and age of infection across 12 European countries before the introduction of routine vaccination, 2007/08 to 2012/13. Eurosurveillance. 2016;21.

4. Bwogi J, Malamba S, Kigozi B, Namuwulya P, Tushabe P, Kiguli S, et al. The epidemiology of rotavirus disease in under-five-year-old children hospitalized with acute diarrhea in central Uganda, 2012-2013. Arch Virol. 2016;161(4):999-1003.

5. Mayindou G, Ngokana B, Sidibé A, Moundélé V, Koukouikila-Koussounda F, Christevy Vouvoungui J, et al. Molecular epidemiology and surveillance of circulating rotavirus and adenovirus in Congolese children with gastroenteritis. Journal of Medical Virology. 2016;88:596-605.

6. Platts-Mills JA, Babji S, Bodhidatta L, Gratz J, Haque R, Havt A, et al. Pathogen-specific burdens of community diarrhoea in developing countries: A multisite birth cohort study (MAL-ED). The Lancet Global Health. 2015;3.

7. De Grazia S, Bonura F, Colomba C, Cascio A, Di Bernardo F, Collura A, et al. Data mining from a 27-years rotavirus surveillance in Palermo, Italy. Infection, Genetics and Evolution. 2014;28:377-84.

8. Chiang GPK, Nelson EAS, Pang TJHS, Law SK, Goggins W, Chan JYC, et al. Rotavirus incidence in hospitalised Hong Kong children: 1 July 1997 to 31 March 2011. Vaccine. 2014;32:1700-6.

9. Kotloff KL, Nataro JP, Blackwelder WC, Nasrin D, Farag TH, Panchalingam S, et al. Burden and aetiology of diarrhoeal disease in infants and young children in developing countries (the Global Enteric Multicenter Study, GEMS): A prospective, case-control study. The Lancet. 2013;382:209-22.

10. Estévez A, Arvelo W, Hall AJ, López MR, López B, Reyes L, et al. Prevalence and genetic diversity of norovirus among patients with acute diarrhea in Guatemala. Journal of Medical Virology. 2013;85:1293-8.

11. Benhafid M, Elomari N, Elqazoui M, Meryem AI, Rguig A, Filali-Maltouf A, et al. Diversity of rotavirus strains circulating in children under 5 years of age admitted to hospital for acute gastroenteritis in morocco, june 2006 to may 2009. Journal of Medical Virology. 2013;85:354-62.

12. Denno DM, Shaikh N, Stapp JR, Qin X, Hutter CM, Hoffman V, et al. Diarrhea etiology in a pediatric emergency department: A case control study. Clinical Infectious Diseases. 2012;55:897-904.

13. González GG, Liprandi F, Ludert JE. Molecular epidemiology of enteric viruses in children with sporadic gastroenteritis in Valencia, Venezuela. Journal of Medical Virology. 2011;83:1972-82.

14. Latipov R, Utegenova E, Kuatbayeva A, Kasymbekova K, Abdykarimov S, Juraev R, et al. Epidemiology and burden of rotavirus disease in Central Asia. International journal of infectious diseases : IJID : official publication of the International Society for Infectious Diseases. 2011;15:e464-9.

15. de Rougemont A, Kaplon J, Fremy C, Legrand-Guillien MC, Minoui-Tran A, Payan C, et al. Clinical severity and molecular characteristics of circulating and emerging rotaviruses in young children attending hospital emergency departments in France. Clinical Microbiology and Infection. 2016;22:737.e9-.e15.

16. Bodhidatta L., McDaniel P., Sornsakrin S., Srijan A., Serichantalergs O. Case-control study of diarrheal disease etiology in a remote rural area in western Thailand. American Journal of Tropical Medicine and Hygiene. 2010;83:1106-9.

17. Cunliffe NA, Ngwira BM, Dove W, Thindwa BD, Turner AM, Broadhead RL, et al. Epidemiology of rotavirus infection in children in Blantyre, Malawi, 1997-2007. J Infect Dis. 2010;202 Suppl:S168-74.

18. Page N, Pager C, Steele AD. Characterization of Rotavirus Strains Detected in Windhoek, Namibia during 1998–1999. The Journal of Infectious Diseases. 2010;202:S162-S7.

19. Zaman K, Yunus M, Faruque ASG, Arifeen SE, Hossain I, Azim T, et al. Surveillance of rotavirus in a rural diarrhoea treatment centre in Bangladesh, 2000-2006. Vaccine. 2009;27.

20. Kang G, Arora R, Chitambar Shobha D, Deshpande J, Gupte MD, Kulkarni M, et al. Multicenter, Hospital‐Based Surveillance of Rotavirus Disease and Strains among Indian Children Aged &lt;5 Years. The Journal of Infectious Diseases. 2009;200:S147-S53.

21. Karsten C, Baumgarte S, Friedrich AW, Von Eiff C, Becker K, Wosniok W, et al. Incidence and risk factors for community-acquired acute gastroenteritis in north-west Germany in 2004. European Journal of Clinical Microbiology and Infectious Diseases. 2009;28:935-43.

22. Wildi-Runge S, Allemann S, Schaad UB, Heininger U. A 4-year study on clinical characteristics of children hospitalized with rotavirus gastroenteritis. European Journal of Pediatrics. 2009;168:1343-8.

23. Sugata K, Taniguchi K, Yui A, Miyake F, Suga S, Asano Y, et al. Analysis of Rotavirus Antigenemia and Extraintestinal Manifestations in Children With Rotavirus Gastroenteritis. PEDIATRICS. 2008;122:392-7.

24. Klein EJ, Boster DR, Stapp JR, Wells JG, Qin X, Clausen CR, et al. Diarrhea etiology in a Children's Hospital Emergency Department: a prospective cohort study. Clinical infectious diseases : an official publication of the Infectious Diseases Society of America. 2006;43:807-13.

25. Grimwood K, Huang QS, Cohet C, Gosling IA, Hook SM, Teele DW, et al. Rotavirus hospitalisation in New Zealand children under 3 years of age. Journal of Paediatrics and Child Health. 2006;42:196-203.

26. Moulin F, Marc E, Lorrot M, Coquery S, Sauve-Martin H, Ravilly S, et al. [Hospitalization for acute community-acquired rotavirus gastroenteritis: a 4-year survey]. Arch Pediatr. 2002;9:255-61.

27. Coulson BS, Grimwood K, Masendycz PJ, Lund JS, Mermelstein N, Bishop RF, et al. Comparison of rotavirus immunoglobulin A coproconversion with other indices of rotavirus infection in a longitudinal study in childhood. Journal of Clinical Microbiology. 1990;28:1367-74.

28. Yeung KHT, Tate JE, Chan CC, Chan MCW, Chan PKS, Poon KH, et al. Rotavirus vaccine effectiveness in Hong Kong children. Vaccine. 2016.

29. Wandera EA, Mohammad S, Komoto S, Maeno Y, Nyangao J, Ide T, et al. Molecular epidemiology of rotavirus gastroenteritis in Central Kenya before vaccine introduction, 2009–2014. Journal of Medical Virology. 2017;89:809-17.

30. Kar S, Pattnaik D, Das SC, Pharveen I. Prospective hospital-based surveillance to estimate the burden of rotavirus gastroenteritis in children less than five years in Bhubaneswar. Clinical Epidemiology and Global Health. 2014;2:52-5.

31. Kiulia NM, Nyaga MM, Seheri ML, Wolfaardt M, Van Zyl WB, Esona MD, et al. Rotavirus G and P types circulating in the eastern region of Kenya: Predominance of G9 and emergence of G12 genotypes. Pediatric Infectious Disease Journal. 2014;33.

32. Motamedifar M, Amini E, Talezadeh Shirazi P. Frequency of Rotavirus and Adenovirus Gastroenteritis Among Children in Shiraz, Iran. Iranian Red Crescent Medical Journal. 2013;15:729-33.

33. Najafi A., Najafi S., Vahdat K., Kargar M., Javdani N. Importance of viral pathogens in children with acute gastroenteritis in the south of Iran. Annals of Saudi Medicine. 2013;33:124-9.

34. Sudarmo SM, Shigemura K, Athiyyah AF, Osawa K, Wardana OP, Darma A, et al. Genotyping and clinical factors in pediatric diarrhea caused by rotaviruses: One-year surveillance in Surabaya, Indonesia. Gut Pathogens. 2015;7.

35. Uzoma EB, Chukwubuikem C, Omoyibo E, Tagbo O. Rota virus genotypes and the clinical severity of Diarrhoea among children under 5 years of age. Niger Postgrad Med J. 2016;23(1):1-5.

36. Zhang S, Yin J, Yang J, Tian L, Li D, Zhang Q, et al. Epidemiology and genetic diversity of group A rotavirus in acute diarrhea patients in pre-vaccination era in southwest China. Journal of Medical Virology. 2017;89:71-8.

37. O'Reilly CE, Jaron P, Ochieng B, Nyaguara A, Tate JE, Parsons MB, et al. Risk factors for death among children less than 5 years old hospitalized with diarrhea in rural Western Kenya, 2005-2007: A cohort study. PLoS Medicine. 2012;9.

38. Díaz J, Pérez Rodríguez A, Olea A, Gonzalez C, Galeno H, Soto F, et al. Sistema de Vigilancia de Rotavirus en Hospitales Centinelas en Chile. Rev Cubana Med Trop. 2009;61:5-12.

39. Nokes DJ, Abwao J, Pamba A, Peenze I, Dewar J, Maghenda JK, et al. Incidence and clinical characteristics of group A rotavirus infections among children admitted to hospital in Kilifi, Kenya. PLoS Med. 2008;5(7):e153.

40. Podkolzin AT, Fenske EB, Abramycheva NY, Shipulin GA, Sagalova OI, Mazepa VN, et al. Hospital‐Based Surveillance of Rotavirus and Other Viral Agents of Diarrhea in Children and Adults in Russia, 2005–2007. The Journal of Infectious Diseases. 2009;200:S228-S33.

41. Seheri LM, Page N, Dewar JB, Geyer A, Nemarude AL, Bos P, et al. Characterization and Molecular Epidemiology of Rotavirus Strains Recovered in Northern Pretoria, South Africa during 2003–2006. The Journal of Infectious Diseases. 2010;202:S139-S47.

42. Satter SM, Gastanaduy PA, Islam K, Rahman M, Rahman M, Luby SP, et al. Hospital-based surveillance for rotavirus gastroenteritis among young children in Bangladesh: Defining the potential impact of a rotavirus vaccine program. Pediatric Infectious Disease Journal. 2017;36:168-72.

43. Mladenova Z, Steyer A, Steyer AF, Ganesh B, Petrov P, Tchervenjakova T, et al. Aetiology of acute paediatric gastroenteritis in Bulgaria during summer months: Prevalence of viral infections. Journal of Medical Microbiology. 2015;64:272-82.

44. Mehendale S, Venkatasubramanian S, Girish Kumar CP, Kang G, Gupte MD, Arora R. Expanded Indian national rotavirus surveillance network in the context of rotavirus vaccine introduction. Indian Pediatrics. 2016;53:575-81.

45. Ianiro G, Delogu R, Fiore L, Monini M, Ruggeri FM, Pagani E, et al. Group A rotavirus genotypes in hospital-acquired gastroenteritis in Italy, 2012–14. Journal of Hospital Infection. 2017;96:262-7.

46. Velázquez FR, Matson DO, Calva JJ, Guerrero ML, Morrow AL, Carter-Campbell S, et al. Rotavirus Infection in Infants as Protection against Subsequent Infections. New England Journal of Medicine. 1996;335:1022-8.

47. WHO. Rotavirus laboratory network. Available at: <http://www.who.int/immunization/monitoring_surveillance/burden/laboratory/Rotavirus/en/> [Accessed 31st August 2018]. 2018.

1. 1 study from the Seychelles and 2 from Hong Kong. The Seychelles is the only country in the AFRO region in the Low or Very Low mortality strata. [↑](#footnote-ref-1)
